# Private garden uses and associated mental well-being benefits during the first UK Covid-19 lockdown – a social media investigation

**DOI:** 10.1371/journal.pone.0289446

**Published:** 2026-04-08

**Authors:** Robert L. Feller, Claire L. Narraway, David FRP Burslem, Laura Colucci-Gray, CGE (Toos) Van Noordwijk, René Van der Wal

**Affiliations:** 1 School of Biological Sciences, University of Aberdeen, Aberdeen, Scotland, United Kingdom; 2 Earthwatch Europe, Oxford, England, United Kingdom; 3 Moray House School of Education and Sport, University of Edinburgh, Scotland, United Kingdom; 4 Earthwatch Europe, Zaltbommel, Gelderland, Netherlands; 5 Department of Ecology, Swedish University of Agricultural Sciences (SLU), Uppsala, Sweden; University of Central Lancashire, UNITED KINGDOM OF GREAT BRITAIN AND NORTHERN IRELAND

## Abstract

During the UK’s first national Covid-19 lockdown in spring 2020, people had greatly diminished access to public green spaces due to government restrictions. This provided an opportunity to investigate people’s discourse on the uses of private green spaces and associated mental well-being benefits during a period of crisis. We used Twitter posts, and thus self-reporting, to identify private garden uses, changes therein during Covid-19 lockdown and associations with mental well-being benefits. Our Twitter search (now X) for garden-related tweets concerned Greater London. Posts written during the first UK lockdown in 2020 and the preceding year’s corresponding period were queried and compared. We subjected all resulting tweets (8,866) to a word count analysis. Next, we iteratively screened posts for false positives and subjected verified posts to thematic analysis until reaching saturation, assigning themes for reported garden uses and mental well-being benefits to 600 relevant posts. The estimated number of tweets mentioning private garden use was 5.2 times higher during the Covid-19 lockdown, confirming the importance of gardens during this period of crisis. We identified five garden use types, of which *socialising and leisure activities* had the greatest relative share of tweets during lockdown (up from 34% to 42%). Communicated garden use also diversified, with *home-based working* and *DIY* expressed almost exclusively during lockdown. Furthermore, the share of posts mentioning garden use to provide mental well-being benefits increased considerably (from 4% to 20%). We identified five categories of mental well-being benefits, with two (*mitigation of restlessness* and *providing hope*) reported only during lockdown. Our findings show that the Covid-19 crisis transformed the way people talked and thought about their private gardens. We suggest that urban planners and conservation organisations reconsider private garden use interests as diverse and changeable and create future programmes to promote multi-functional private gardens for healthier communities.

## Introduction

Urban green spaces provide important mental well-being and physical health benefits for people, including recovery from stress [1,2]. Such benefits are especially strong when green spaces are visited over a prolonged period of time [3] and when they display a high biodiversity [4,5]. Among the different types of urban green space, private gardens are an important resource for people and wildlife [6]. Primary motivations for private garden use include an interest in nature, appreciation of outdoor green space for social activities and engagement in gardening [7–9]. How such diversity of garden uses connects to specific mental well-being benefits remains poorly understood. As cities become increasingly densely populated and built-up, such knowledge is important for informing the development of urban green space policies and conservation programmes to prioritise and ensure effective use of small green spaces for mental well-being [10,11].

Urban green spaces, including private gardens and parks, are often used differently during social crises [12–14]. Such changes are driven by personal motivations and in response to new regulations and campaigns [15–17]. For example, urban green space in the UK was increasingly used for growing food, socialising and supporting mental well-being during the Second World War [18]. The most recent and acute social crisis to affect the UK, and much of the world, was the Covid-19 crisis [19]. Governments around the globe introduced mandated lockdowns that severely reduced people’s freedom of movement (S1 Fig), which led many people to change their use of urban green space. Some public areas, such as streets lined with trees in Israel, Croatia and Spain, saw moderate increases in use during national Covid-19 lockdowns, while visitations to parks (which were restricted in many countries) declined strongly in these countries compared to pre-Covid-19 levels [20]. Parks remained open in the UK but were also used less often during the first Covid-19 lockdown [21], with residents expressing concerns about visiting public spaces because of perceived heightened infection risk [22,23]. By contrast, private gardens in the UK, as in other countries such as Canada and Italy, saw an increase in use during the Covid-19 crisis [20,24,25]. Such increased relevance of gardens provides a good opportunity to investigate the diversity of garden uses and their associated benefits for mental well-being. An estimated 82% of people in the UK with a private garden made use of it at least weekly during the first Covid-19 lockdown [25].

Covid-19 restrictions caused adverse effects on mental well-being, with an increase of 13.5 percentage points in the prevalence of mental well-being problems in the UK in April 2020, compared to 2017−2019 [26]. Limitations on social contact led to a general decline in mental well-being indicators [27–29]. Interaction with urban green space was one factor that mitigated the decrease in mental well-being during lockdowns [30] through a reduction in symptoms of depression and a raised frequency of positive emotions [30–33]. Urban green spaces acted as catalysts for mental and physical health [33]. Out of several categories of urban green space, private gardens appear to have been most effective at mitigating mental distress during the Covid-19 crisis, such as in Rio de Janeiro, Brazil [34] and other countries with similarly rigorous lockdowns [35], including the UK.

One way to further understanding of the diversity of garden uses and attributed health benefits is to investigate their representation – during a time when they clearly matter – on social media, as was done for balconies during the 2020 Covid-19 lockdown in Spain, demonstrating their (temporary and partial) substitution of public space [36]. Investigating such discourse, we ask the following questions: (1) how did the extent and portrayal of private garden use change in response to the introduction of Covid-19 lockdown; and (2) to what extent did self-reported benefits for mental well-being coincide with private garden uses during the time of the Covid-19 restrictions?

## Methods

### Approach

We used data collected from a third-party source, i.e. the micro-blogging platform Twitter [37]. Twitter (now X) provides access to large amounts of data from material posted in the moment, as people readily share experiences based on motivations such as interpersonal communication [38]. Furthermore, Twitter provides easy, systematic access to its data through an application programmable interface (API), which is not available for other popular microblogging platforms such as Facebook [39]. Twitter users are believed to show a high degree of self-disclosure in posts, particularly where users have strong (friendship or other) relationships [40]. Yet, Twitter posts are written to project a certain image and to generate certain reactions and thus are best seen as discourse [41]. Within that constraint, we developed a methodology to reveal change in how people talked about their private gardens during Covid-19 restrictions.

We selected tweets originating from an area that spans a circle with a 32 km (20 mile) radius centred on central London (51.528°, −0.228°), resembling roughly the area of Greater London. This area, containing an estimated 3.5 million households, was chosen because it accommodates a wide range of socio-economic groups living in close proximity [42–44]. Indeed, within this area are two London boroughs (Hackney and Haringey) that rank amongst the 20 most deprived areas of England, as well as boroughs that regularly rank amongst the wealthiest, including Richmond and Kingston [45,46]. Less than 79% of households have access to a private or shared garden, which is below the national average (88%) [47,48]. About 23% of Greater London is private or shared, domestic garden land and 14% is estimated to be vegetated garden greenspace [49].

Ethical approval was obtained for this study through the University of Aberdeen Institutional Review Board. All Twitter posts were publicly available. Following the Twitter (X) data use policy, we do not publish raw data, ensuring the anonymity of the posts’ authors [50], but only present Twitter IDs in the dataset (S7 Data).

### Data collection

We extracted Twitter posts from 24 March to 30 April in 2020 to obtain our ‘first Covid-19 lockdown sample’ and from 24 March to 30 April in 2019 to obtain a ‘pre-lockdown control sample’ for comparison. By selecting the same time period (and hence similar as possible seasonality), the year before (and hence nearest to the focal period), while there was no social crisis at national scale, we deemed this period the most reasonable representation of non-Covid times. To gain further confidence about this choice, we investigated whether this period was typical in terms of Twitter compared to earlier years. We captured tweets from the start of the spring gardening season, which is usually in March [51]. There were no marked differences in Twitter activity (weekly counts of garden-related tweets in the study area) across the relevant months between the three pre-lockdown years (2017–2019), ruling out any general increase or decrease across the pre-lockdown years (S2 Fig). We also did not find evidence for Twitter activity to be related to weekly average temperature – another potential confounding factor (S3 Fig). Therefore, our pre-lockdown sample seemed indeed an appropriate choice.

**Fig 1:**
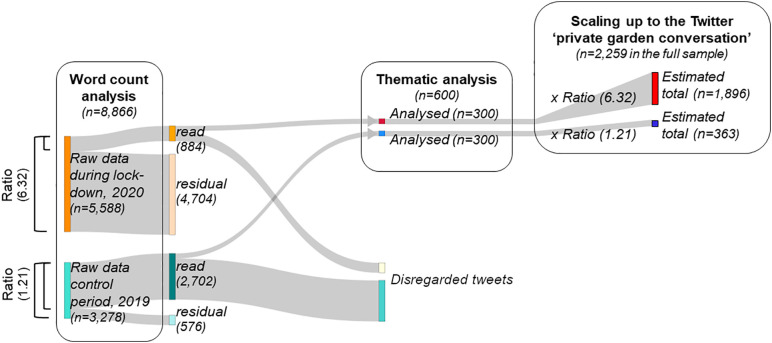
Schematic illustration of the data volumes for our two analyses and the respective sampling process. Our Twitter search via the Full-Archive API, performed for the keyword string, geo-location (Greater London, UK) and two periods (lockdown in Spring 2020 and control in Spring 2019), yielded a total of 8,866 posts. We used all of those for our Word count analysis. A subset from each period was then read and all those referring to ‘private garden’ were included in the subset used for our Thematic analysis (n = 300 per period). Ratio refers to the number of tweets at the onset divided by the number of read tweets (‘*raw data* to *read tweets*’ ratio) and was used to scale up to the respective Twitter ‘private garden conversation’ in the total sample (n = 8,866; Fig 1).

Posts were retrieved using the Twitter Full-Archive Application Programmable Interface (API) v1, facilitated by *Python* 3.7.6 [52] and the *searchtweets* 1.7.6 library [53]. All tweets for the specified keyword string “*garden OR gardened OR gardening*”, the geographical scope and the two time frames were collected for this study (Fig 1). All 8,866 posts held on Twitter’s archive for that search configuration were collected, of which 5,588 belonged to the lockdown period in 2020 and 3,278 to the control period in 2019, and the complete dataset was used for Word count analysis (see below).

### Word count analysis

We performed a word count analysis [54] on the total sample of 8,866 tweets for methods triangulation, to assess differences in the occurrence of keywords between the 2020 lockdown period and corresponding 2019 control period concerning private garden use and associated benefits for mental well-being. The data from each sample period was processed using the *Python* 3.7.6 library [52] and the natural language toolkit *NLTK* 3.6.2 library [55]. Subsequently, during the process of data cleaning, tweets underwent tokenisation, the process of splitting text into words, and the removal of stop words [56]. To remove stop words (common words such as ‘about’ and ‘before’) from the dataset, we deployed the *NLTK* library’s *stopwords* function [55]. Emojis in tweets were transformed from their symbolic form to text to make their meaning accessible for textual analysis, for which we used *Python* 3.7.6 and the *demoji* 1.1.0 library [52,57].

The word count analysis yielded a list of individual words ranked by their frequency for each period. We also determined prevalent phrases (consisting of two or three words, i.e. 2-grams and 3-grams) by applying the *ngrams* function of the *NLP* library [58] in R v. 4.3.0 [59] for both periods. From this, a list of 150 top-ranking words or phrases was compiled (see Table 1 for the top 20). To further improve the interpretive power of the word count analysis, words and (2-gram and 3-gram) phrases with similar meanings were attributed to groups, of which two were relevant to this analysis: ‘Private garden’ and ‘General benefit for mental well-being’. However, during the word count analysis, it became apparent that an additional group was required for words that referred to ‘Public space’. All other words and word groups (in the top 150) were placed under ‘Ambiguous’, as unclear whether belonging to any of the other three groups.

**Table 1:** Keywords and phrases identified as ranking highest (top 150) in the word count analysis of tweets, grouped by topic (Private garden, General benefit for mental well-being, Public space, Ambiguous).

Topic	Keywords and phrases	Frequency during lockdown period, 2020	Frequency during control period, 2019
**Private garden**	gardening (ranked as #5), home (#23), back garden (#24), house (#32), front garden (#125), neighbours (#133)	1,030 (344 + 200 + 199 + 137 + 67 + 83)	316 (160 + 43 + 41 + 53 + 19 + 7)
**General benefit for mental well-being**	lovely (#18), beautiful (#19), love (#20), sun (#37), happy (#47), sunshine (#49), thanks (#62), thank (#64), hope (#86), enjoying (#100), sunny (#116), lucky (#123)	1,319 (158 + 153 + 151 + 117 + 109 + 115 + 110 + 102 + 87 + 72 + 66 + 79)	630 (103 + 107 + 109 + 63 + 46 + 40 + 34 + 39 + 24 + 29 + 27 + 9)
**Public space**	Covent (#4), Covent garden (#7), park (#38), Covent garden London (#46), sky garden (#78), Kew (#81), beer garden (#99), secret garden (#101)	351 (70 + 68 + 95 + 7 + 14 + 18 + 42 + 37)	1,410 (438 + 410 + 83 + 149 + 108 + 97 + 61 + 64)
**Ambiguous**	garden (#1), London (#2), day (#3), just (#6), today (#8), gardens (#9), back (#10), one (#11), now (#12), time (#13), can (#14), good (#15), like (#16), get (#17), will (#21), new (#22), lockdown (#25), spring (#26), morning (#27), garden London (#28)	16,446 (3943 + 360 + 365 + 324 + 331 + 185 + 310 + 235 + 260 + 240 + 232 + 205 + 193 + 202 + 170 + 137 + 237 + 107 + 178 + 48)	8,786 (2198 + 654 + 160 + 164 + 141 + 231 + 97 + 123 + 91 + 87 + 75 + 88 + 82 + 70 + 81 + 110 + 0 + 127 + 55 + 185)

For the words and phrases under ‘Ambiguous’ only the 20 highest-ranking ones in that category are presented. The rank order of each keyword or phrase in the total sample of tweets is across both periods (second column), while frequency is listed for each period separately (third and fourth columns).

### Pre-selecting relevant data, generating codes and identifying themes

We subsequently started reading tweets to identify a subset for thematic analysis. To ascertain that tweets referred to actual use of a private garden, we set the following criteria: (i) the tweet refers to the author’s existing (or desired) private garden; and (ii) the tweet describes an interaction with that private garden. We read batches of 50 randomly selected tweets and alternated between both periods to avoid systematic bias. We disregarded any tweets that were irrelevant (Fig 1), e.g. when a post referred to any place or context that contained the term ‘garden’ without it being a private garden (Table 2). Relevant tweets (i.e. fulfilling the two criteria listed above) were immediately marked up with initial codes (S4 Table), which we then used to identify emerging themes [60]. Where included in (or linked to) the tweets, we also reviewed images to ensure accurate interpretation and coding. We repeated this until no further codes or anticipated themes were detected, following the guiding principle of saturation [61]. We identified 300 tweets from each time period (600 tweets in total). This required reading 3,586 tweets in total. Tweets were much more frequently assessed as relevant in the Covid-19 lockdown sample (300 out of 884 read tweets) than in the control sample (300 of 2,702 read tweets) (Fig 1). Tweets not utilised in thematic analysis after reaching saturation remained as residual: 4,704 for the Covid-19 lockdown period and 576 for the control period (Fig 1). The lengths of tweets selected for thematic analysis were comparable between the two data periods.

**Table 2:** Reasons why posts were detected as false positives during the initial screening of the data.

Reasons for posts’ detection as false positives
unrelated – public space unrelated – public garden / park unrelated – real estate commercial unrelated – other commercial unrelated – not about post author’s own private garden but gardens in general unrelated – touristic posting unrelated – garden on business premises (not private) unrelated – beer garden (of a restaurant) unrelated – community garden (not private) unrelated – allotment garden (not private) unrelated – other

The overall sample of 600 tweets was derived from 478 people. We allowed for the inclusion of multiple tweets from individuals as this accommodates different self-reporting behaviours [62]. Individuals typically occurred once in our sample (77% and 68% of the cases for the Covid-19 lockdown and control period, respectively) and for one person to occur with more than 2 posts was rare (S5 Fig). Fifteen users had posts in both periods. Given these distributions, the influence of single individuals on the overall findings will have been limited.

### Thematic analysis steps

To detect patterns of meaning in the data, the sample of (600) tweets was subjected to thematic analysis [63]. Five phases were performed, in part recursively: (1) familiarisation with the data, (2) generation of initial codes (S4 Table), (3) searching for themes, (4) reviewing of themes and (5) theme definition and analysis [60]. The word count analysis informed phases 1 and 2 (unfolded in the previous section), as it helped us to understand the data and anticipate initial codes. Themes (phase 3) were identified abductively, i.e. through parallel engagement with empirical data (including through the initial coding) (S4 Table) and understanding from the literature (notably on private garden use and mental health benefits) [64]. By using this approach, it was possible to detect latent (underlying) and manifest (explicitly mentioned) notions effectively in the thematic analysis [63], thus ensuring that the results are rooted in both the existing knowledge and the raw data [65]. We reviewed the themes (phase 4) by re-reading our comments containing suggestions for specific themes annotated to relevant tweets in earlier phases; this did not lead to marked changes in the themes. We then defined and named the themes (Tables 3,4), revisited all (600) tweets, and assigned to each tweet the private garden use theme and – where applicable – the benefit for mental well-being that dominated its content (phase 5).

**Table 3:** Identified themes of private garden use, with descriptions. All but one (*Wanting a garden*) occurred in the literature.

Private garden use theme	Description
*Socialising and leisure activities*	Leisure activities that are often conducted indoors but here outdoors, in the private garden [66] (e.g. having food/beverages, leisure reading); can involve socialising (e.g. giving each other company, chatting) with family members, friends and pets [9]
*Ornamental and vegetable gardening*	Activities to garden for aesthetics (e.g. beauty, neatness) [67] or to grow food (vegetables, fruit) [9]. We grouped these activities because many home gardens integrate ornamental and edible plants, and social media posts did not provide sufficient contextual information to consistently and reliably distinguish between aesthetic and productive gardening intentions
*Wildlife-friendly activities*	Activities where plants and animals in the garden are valued [8,9], often including active engagement [68] such as watching or photographing birds or butterflies [69]; activities that create habitat or infrastructure for biodiversity (e.g. nest boxes, planting for pollinators) [70]
*Wanting a garden*	Reflects a desire to have or use a garden at home; relevant to include in this study as well as a (virtual) interaction with a (potential) private garden, as mental well-being benefits can be connected to imagining a positive future, associated with optimism and vivid mental imagery [71]
*DIY (do-it-yourself building and maintenance)*	Represents activities such as erecting structures (e.g. sheds, fences, patios) or other objects in the private garden (such as garden furniture) and their upkeep
*Home-based work*	Involves conducting (office-related) work from the private garden or its vicinity but also obtaining inspiration for work in the private garden; for some residents, it is performed inside a dedicated sheltered space in the garden, such as a summerhouse [72]

**Table 4:** Identified themes of private garden use-related benefits for mental well-being, with descriptions and origins.

Mental well-being benefit theme	Description
*Mitigation of restlessness*	Describes the lowering of a state of anxiety and uneasiness, primarily through being ‘positively distracted’ by sights or sounds of garden features (e.g. running water, leaves of trees) in a private garden [73]; agitation is also lowered by slowly moving (wandering) through a garden [74]
*Thankfulness*	Using gratitude to reflect on life and plans positively [75]; *thankfulness* has a healing effect on the individual when experienced and expressed actively and consciously in the form of a practice [76]; known to imbue individuals with higher life satisfaction and social feelings [77]
*Uplifts*	Characterised by an experience of joy and elation [78], which can arise, e.g. in association with observing plants grow or may include a spiritual dimension [79]; may also relate to the garden as a refuge [80]
*Providing hope*	Describes fostering the capability to envision pathways and the ability to motivate oneself to follow them [81]; is associated with improved mental well-being for adults, in part linked to an improved ability to cope with stressful events [82,83]
*Contemplation*	Emerges from various practices (including meditation) associated with improving mental well-being [84]; green spaces containing trees, including private gardens, are often chosen for contemplative practices because trees evoke emotional responses and become the object of contemplation and commemoration [85]

We defined six themes of private garden use: *socialising and leisure activities*; *wildlife-friendly activities*; *ornamental and vegetable gardening*; *wanting a garden*; *do-it-yourself (DIY)*; and *home-based work* (Table 4). Furthermore, we defined five themes of mental well-being benefits related to private garden use: *mitigation of restlessness*; *thankfulness*; *uplifts*; *providing hope*; and *contemplation* (Table 4).

### Scaling and contextualising

Scaling up the thematic analysis of the identified tweets to the conversation across the total sample of extracted tweets required starkly different ratios, namely 6.32 (5,588/884) and 1.21 (3,278/2.702) for the lockdown and control period, respectively (Fig 1). We will henceforth refer to these as *raw data* to *read tweets* ratios. The different ratios for both periods suggest that the share of private garden use was much higher for Twitter posts from the study area during the Covid-19 lockdown period than the respective 2019 period. Resultant count data of garden uses and benefits for mental well-being were multiplied by the *raw data* to *read tweets* ratios (6.32 for the Covid-19 lockdown and 1.21 for the 2019 period) to scale our thematic analysis sample to represent the magnitude of the Twitter ‘private garden conversation’ in the total sample. Furthermore, quotes were identified by re-reading the tweets with their assigned themes (regarding private garden use and mental well-being benefits) to illustrate the nature of change in response to lockdown, contextualising the study’s primary results.

### Statistical analysis

Chi-square tests were used to examine differences between Twitter data from focal lockdown (2020) and control (2019) periods regarding: (1) the frequencies of keywords and phrases within topics (in the word count analysis); (2) counts of tweets expressing different private garden use themes (in the thematic analysis); and (3) the distribution of private garden use themes (in the thematic analysis). We ran the tests in *Python* 3.6.8 [52] with the library *SciPy* 1.5.4 [86].

Furthermore, Twitter posts were grouped into posts with and without benefits for mental well-being and compared for the Covid-19 lockdown period (2020) and the control period (2019), distinguished by garden use. First, the absolute estimated total number of tweets were plotted and compared between the periods. Second, to better illustrate the relative increases in posts with reported benefits for mental well-being, estimated total number of tweets for each garden use were transformed to percentages per total tweets across both periods. We simplified the presentation of the results by summarising tweets without reported benefits for mental well-being, not distinguishing by garden use, as one relative total share per each period.

Another line of investigation was to compare the estimated total number of tweets for each mental well-being benefit, which we did by visual comparison based on an overview figure (Fig 4). Additionally, we distinguished the benefits for mental well-being reported per garden use and compared these in their estimated total number of tweets for each of these specifically (based on Fig 5) and again compared these between the periods of Covid-19 lockdown (2020) and the control year (2019).

## Results

Across our study area, 5,588 tweets referencing gardens or gardening were posted during lockdown, compared to 3,278 tweets during the same period the year before (2019). Based on the *raw data* to *read tweets* ratios that we found for the 2020 lockdown period (6.32) and the 2019 period (1.21), this corresponds to an estimated total of 1,896 tweets about private garden use originating from the study area in the 2020 lockdown period and 363 in the 2019 period (Fig 1). Tweets relating to private gardens were thus around 5.2 times more abundant during the 2020 lockdown period than in the analogous period in 2019. General Twitter activity in the study area during the 2020 lockdown remained at similar levels to those of the same period in 2019, 2018 and 2017 (S6 Fig). Thus, the identified increase was not driven by any changes in general Twitter activity but by private gardens being a more common topic of conversation in tweets written during lockdown.

### Word count analysis results

**Fig 2:**
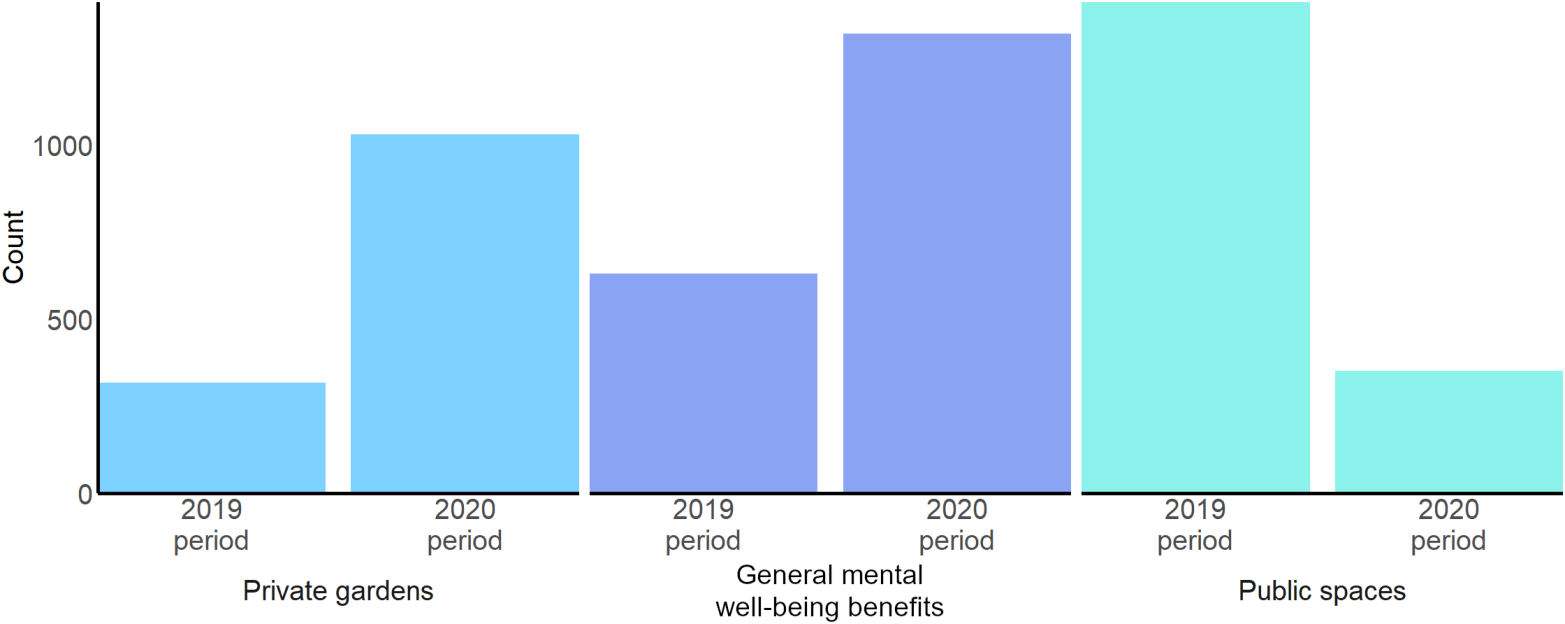
Total number of occurrences (count) of keywords and phrases per identified topic in tweets submitted during lockdown (2020) and the same time interval (i.e. 24 March – 30 April) in 2019. The topics ‘*private gardens*’ and ‘*general benefits for mental well-being*’ were much more common during lockdown, while ‘*public spaces*’ had its highest share in 2019 and thus showing the opposite pattern.

Word count analysis of the total sample of tweets confirmed that keywords and phrases (Table 1, Fig 2) belonging to the topic *‘private gardens’* were referenced significantly more often during lockdown, in 2020, (1,030 counts across tweets) than in 2019 (316 counts) (*χ* ^2^ = 56.8, df = 5, p = 0.001). Mentions of ‘*general benefits for mental well-being’* more than doubled from 2019 (630 counts across tweets) to 2020 (1,319 counts) (*χ* ^2^ = 63.3, df = 11, p = 0.001). Concordantly, far fewer references to *‘public places’* were made in 2020 (351 counts across tweets) than the year before (1,410 counts) (*χ* ^2^ = 222.3, df = 7, p = 0.001).

### Private garden uses – thematic analysis results

**Fig 3:**
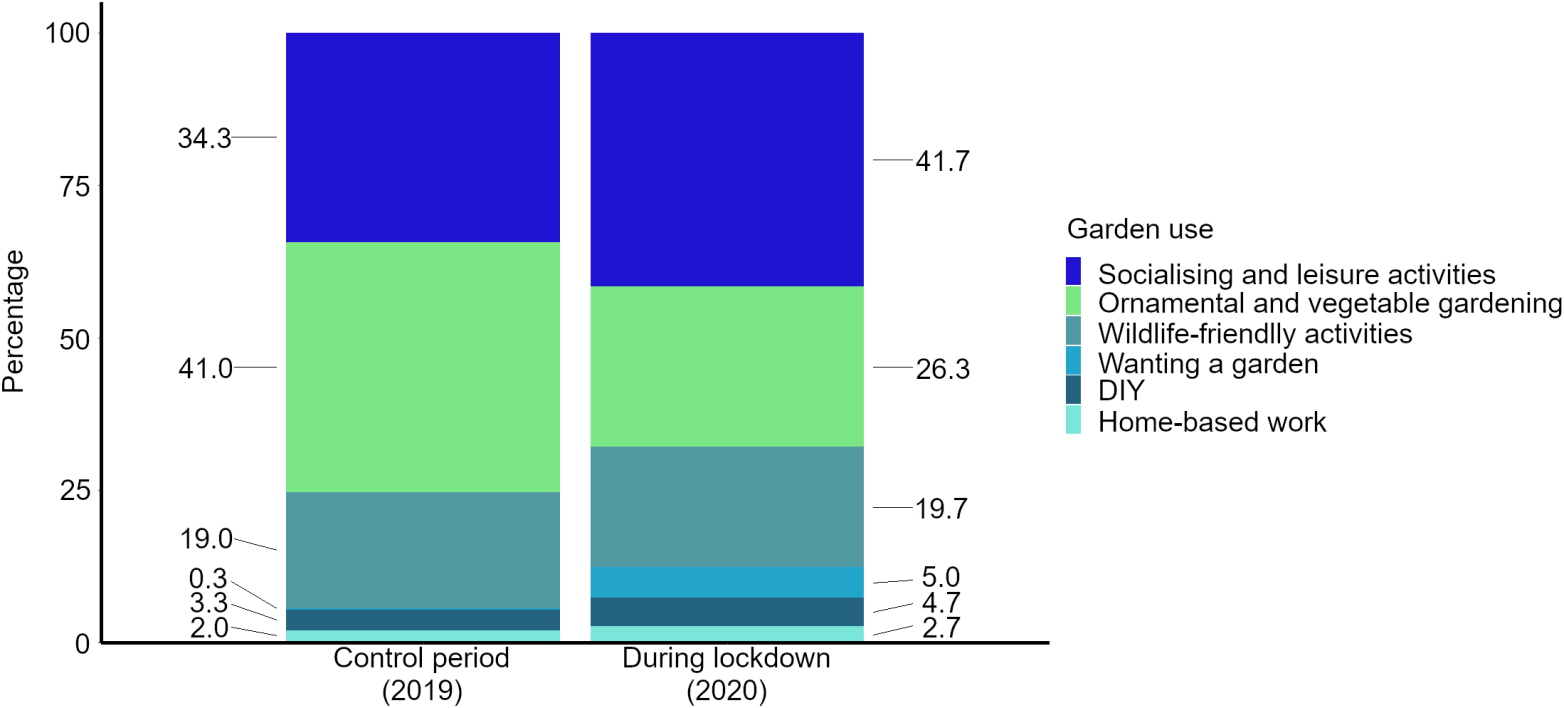
The relative shares of private garden use themes identified from tweets for the control period (2019) and during lockdown (2020). The most substantial changes from 2019 to 2020 (lockdown) were increases in the percentage of tweets concerning *socialising and leisure activities* (up by 7.4% to 41.7%) and a strong concomitant decrease for ones referring to *ornamental and vegetable gardening* (−14.7% to 26.3%). *Wanting a garden* appears during lockdown with a relative share of 5.0%, while this was only 0.3% in the year before.

**Fig 4:**
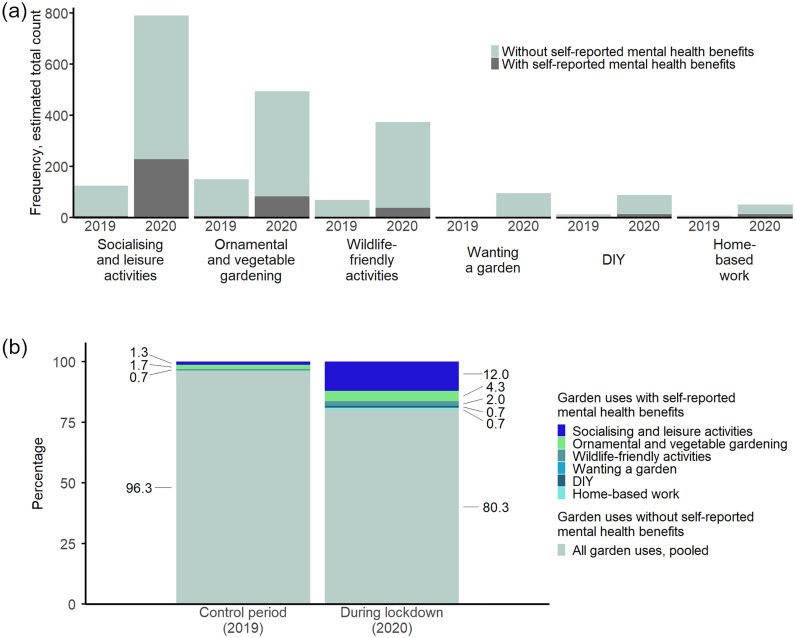
Frequency distribution across all themes and relative abundance of tweets with and without identified benefits for mental well-being. **(a)** Frequency distribution (estimated total number of tweets) across all identified themes, for tweets with (dark grey bars) and without (light-green bars) self-reported benefits for mental well-being. **(b)** Relative abundance of tweets with and without identified benefits for mental well-being, whereby the former is further broken down into the respective garden use themes, revealing proportionally greater benefits for mental well-being from private gardens during lockdown, notably in the context of *socialising and leisure activities*.

From the six private garden use themes that thematic analysis of the subset of 600 tweets produced three (*wanting a garden*, *DIY* and *home-based work*) occurred almost exclusively during lockdown. However, the three private garden uses that were most prominent in 2019 (*ornamental and vegetable gardening, socialising and leisure activities* and *wildlife-friendly activities*) remained so during lockdown in 2020 (Fig 3). All private garden uses were communicated more often in absolute terms in the 2019 period compared to the Covid-19 lockdown in 2020 (Fig 4a). Overall, the distribution of themes between the 2019 and 2020 periods differed significantly (*χ* ^2^ = 21.3, df = 5, p = 0.001).

S*ocialising and leisure activities* showed an increase in frequency between 2019 and 2020 from 34% to 42% in relative terms (Fig 3) and 6.3× in terms of estimated total number of tweets mentioning such activities (Fig 4a). *Socialising and leisure activities* thus became the predominant garden use tweeted about during lockdown, a change from the 2019 period, when *ornamental and vegetable gardening* ranked first. *Ornamental and vegetable gardening* decreased in relative share from 41% to 26% but increased in estimated total number of mentions by a factor of 3.4, making it the second most reported private garden use during Covid-19 lockdown (Fig 3). *Wildlife-friendly activities* had a similar relative share between the two years (19% and 20% for 2019 and 2020, respectively) but increased 5.4× in estimated number of mentions. The theme *wanting a garden* rose sharply in its relative share from 2019 (0.3%) to the 2020 lockdown period (5%), with estimated total number of mentions being 78 × greater. *DIY* garden use saw an increase in relative share from 3% in 2019 to 5% during the 2020 lockdown, corresponding to a 7.3 × greater estimated total count. *Home-based work* increased slightly in share from 2019 (2%) to the lockdown period (2.7%), translating into a 7.0 × greater estimated total count (Fig 4a).

### Benefits for mental well-being – thematic analysis results

**Fig 5:**
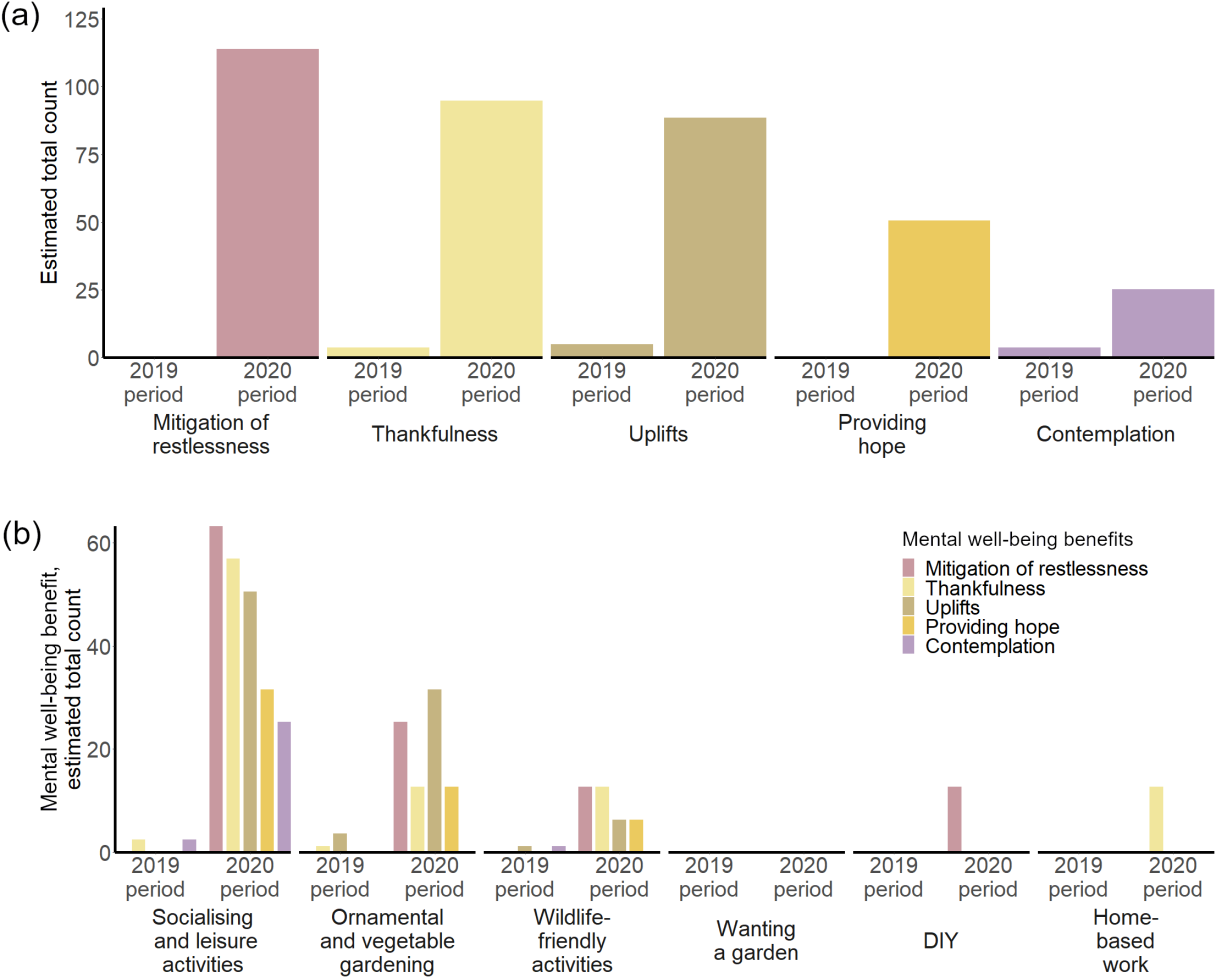
Benefits for mental well-being communicated during lockdown and in the respective 2019 control period. **(a)** Benefits for mental well-being per identified category. The mental well-being categories *mitigation of restlessness* and *providing hope* appear exclusively during lockdown. **(b)** Cross-sectional illustration of benefits for mental well-being per corresponding private garden use type during lockdown (2020) and in the control period (2019). *Socialising and leisure activities* display a diversity of strongly represented self-reported benefits for mental well-being.

Benefits for mental well-being flowing from private gardens were much more frequently referred to during lockdown than in the year before (20% *vs* 4% of tweets, based on the estimated total counts; Fig 4b). We identified benefits for mental well-being that fall into five categories. Three categories (*thankfulness*, *uplifts* and *contemplation*) had low estimated total occurrences in the 2019 period (4, 5 and 4 [out of 3,378 tweets, see Fig 1]) but grew to notably high occurrences during lockdown (95, 89 and 25 [out of 3,278 tweets, see Fig 1]; Fig 5a). The remaining two categories (*mitigation of restlessness* and *providing hope*) were not mentioned during the 2019 period but reached high total estimated occurrences during lockdown (114, 51; Fig 5a).

*Socialising and leisure activities* in private gardens were associated with the highest frequency of self-reported mental well-being benefits of any private garden use during lockdown (Fig 5b), with *mitigation of restlessness*, *thankfulness* and *uplifts* identified as the main benefits (Fig 5b).

Furthermore, *ornamental and vegetable gardening* and *wildlife-friendly activities* also yielded multiple types of self-reported benefits for mental well-being to private garden users during lockdown. For *ornamental and vegetable gardening,* the mental well-being benefits *uplifts* and *mitigation of restlessness* were most regularly mentioned (32 and 25 estimated total number of tweets; Fig 5b), while for *wildlife-friendly activities* the benefits *thankfulness* and *mitigation of restlessness* predominated (both 13 estimated total occurrences; Fig 5b)*.*

*Home-based work* and *DIY* were associated with a single benefit for mental well-being, *thankfulness* and *mitigation of restlessness* respectively (both 13 estimated total occurrences; Fig 5b), and only during Covid-19 lockdown. Given that *wanting a garden* is a desire, rather than a garden use, we did not identify concrete mental well-being benefits to it.

## Discussion

We examined self-reported private garden uses and associated benefits for mental well-being on Twitter during the UK’s first national Covid-19 lockdown and the preceding year for comparison. This first lockdown was a period during which restrictions prevented individuals from leaving their homes except for essential shopping, a daily outdoor exercise session, medical needs and commuting to essential jobs [87]; it was prohibited to meet people from different households [87]. Public parks remained open, but many retail outlets had to close [87]. We found that during this difficult period, private gardens were highly relevant: the estimated total number of tweets about private garden use was 5.2 times higher during the Covid-19 lockdown and the percentage of tweets mentioning benefits for mental well-being of garden use increased from 4% to 20%. In the subsequent sections, we discuss how Twitter users talked and thought about garden usage during lockdown and what this revealed about health benefits associated to private gardens more generally.

### Socialising and leisure activities

*Socialising and leisure activities* became the garden use most frequently tweeted about during lockdown, with an increase in frequency of 7% in relative terms across all garden uses and more than five-fold growth in absolute numbers, likely driven by lockdown having reduced opportunities for people to interact with others, especially outside their homes. The private garden became an important place for socialising activities, partly in pursuit of ways to meet up when restrictions hindered other forms of meeting people (“*BBQ yesterday. A really nice way to enjoy our garden space alongside our neighbours without getting too close!*”). Our findings concur with other studies showing that private gardens are places to spend time alone, with family, pets, friends and neighbours [88–90] and spaces enabling users to participate in leisure activities [91]. Conversations portrayed the garden space also as important for spending time with non-human others such as their pets (“*Doing #lockdown right today. Home-made iced tea […] & sunbathing with the dog. I am so grateful to have a little garden, even if it only gets a few hours sun.”)*, who have been reported to provide companionship, conflict-free relationships, a sense of purpose and ameliorated feelings of loneliness during the Covid-19 crisis [90].

*Mitigation of restlessness* was the most frequently communicated mental well-being benefit associated with this garden use during lockdown (and in general) and facilitated by leisure opportunities that the private garden offered *(“What can I do with my time? #gardening #lockdown #barbecue”).* This mental well-being benefit is highly relevant because restlessness increased during lockdown as people’s everyday routines and lives were put on hold [92,93]. One key mechanism for lowering restlessness is distraction [94], which the organisation and pursuit of socialising activities with family may provide. In addition, exercising and sports in the private garden during lockdown were regularly connected to releasing stress and anxiety (“*Horrible. Shaky and tearful. A jog round the garden helped*”). Indeed, participation in sports activities is known to improve mental well-being through physiological, biochemical and psychological (providing distraction and self-efficacy) processes, with the social aspect of sport (albeit limited during lockdown) also providing an outlet for those suffering from conditions such as depression [95].

Two other benefits for mental well-being strongly associated with *socialising and leisure activities* during Covid-19 lockdown were *thankfulness* and *uplifts.* People reported being thankful for the company of others and for features of the garden and the surrounding natural world (“*Isolation means family time in the garden. Thank God for trees and sky and grass*”). Reports on uplifts experienced by people concerned positive effects on mental well-being, directly (“*A few days in my garden is all the therapy I need. Well garden and gin!*”) or indirectly describing experiences which brightened their mood (“*Sat in the garden in central London and I can hear the birds singing. Quite amazing.”)*. Both *thankfulness* and *uplifts* are helpful coping strategies for overcoming challenges in periods of crisis that build resilience and protect people from developing depressive symptoms [96].

We identified the benefit for mental well-being of *contemplation* in the context of *socialising and leisure activities*, which was also much more frequently communicated during lockdown and regularly included reflective sentiments (“*Sitting in the garden […] you can hear a gentler city tonight. The sirens aren’t as incessant, and I can hear a cat’s meow and the wind in the trees.”)*. This finding concurs with Marsh et al. (2021) indicating that during the Covid-19 lockdown, people’s experiences increasingly included sensory and emotional dimensions, as well as aspects of contemplation and mindfulness [80].

Overall, our findings point to *socialising and leisure activities* in private gardens being of central importance for providing benefits for mental well-being to residents during lockdown and mitigating the effects of reduced physical and social contact [97]. Private gardens mediate between public and private home areas, providing a space for low-threshold interactions with other people, which help build informal support systems, i.e. through day-to-day contact with neighbours and physical activity [98]. This highlights the importance of garden design that ensures and further enhances the capacity of private gardens to benefit mental well-being through enabling *socialising and leisure activities*.

### Ornamental and vegetable gardening

Mentions of *ornamental and vegetable gardening* decreased by 12% in relative share but were still more than three times as high in absolute numbers during lockdown as compared to before and was the second most commonly mentioned private garden use during Covid-19 lockdown. Residents often mentioned vegetable and ornamental gardening types simultaneously (“*[…] I’ve got plenty of time on my hands & hundreds of seeds I’ve saved over the years, both veg & flower”)*, with both involving planting. Substantial media coverage of ornamental and vegetable gardening during the Covid-19 lockdown [99], including reports of gardeners planting a greater diversity of garden plants, crops and varieties [100], suggests an increased interest in these gardening activities.

Increased motivation for vegetable gardening during Covid-19 lockdown to ensure food security through growing more food was communicated (“*Gardeners life […] #GrowYourOwn #gardening […] _leafy green _carrot _broccoli _herbs*”), as well as harvesting of wild food (“*Mum’s leek and nettle #borek made using freshly picked nettles from our garden. […]”).* Vegetable gardening has historically been important to residents in times of crises, e.g. after the collapse of the socialist bloc in Cuba and in World War II [14,101]. Similar efforts towards greater self-sufficiency through gardening were written about during lockdown [102].

The estimated total number of tweets referring to benefits for mental well-being in the context of *ornamental and vegetable gardening* during lockdown showed a substantial increase compared to the control period. *Uplifts* were the most frequently communicated mental well-being benefit (“*Gardening […] It’s a good good good afternoon after the stretch of a particularly hard week.”)*. *Mitigation of restlessness* was also frequently associated with *ornamental and vegetable gardening*, partly characterised by people looking at the Covid-19 lockdown as a situation in which they feel the need – potentially driven by the stress factors of the crisis – to get gardening tasks done (“*Another bright and sunny day... My garden is going to look better than it ever did...”)*. *Thankfulness* was another benefit for mental well-being mentioned relatively frequently for *ornamental and vegetable gardening* during Covid-19 lockdown, in part referring to experiencing beauty (“*More prettiness. So grateful I have a garden right now. [photo with tulips and narcissus]*”). These benefits of *ornamental and vegetable gardening* are also a documented outcome of floral therapy, which uses flowering plants to achieve mental well-being [103]. Additionally, the direct pleasure that gardeners experience from frequent gardening appeared a strong motivation for engagement with private gardens, yielding benefits for mental well-being, presenting a crucial co-benefit [104].

### Wildlife-friendly activities

Self-reported *wildlife-friendly activities* – the third most frequently tweeted about private garden use during lockdown – included wildlife watching (“*Good start to the day. Mute swan added to the lockdown garden list! […]*”) and creating habitat (“*not […] cutting and strimming the grass areas […]. Our early pollinators need the weeds and wild flowers at this time of year”)*. Some people referenced nature conservation organisations’ campaigns during the Covid-19 crisis, such as that initiated by the British Trust for Ornithology (“*[...] @BTO_GBW never thought I would see a red kite over my Ashford estate garden #happyisolatingbirdwatcher”)*. Conservation organisations may have contributed to the abundant engagement in *wildlife-friendly activities*, as several organisations from the UK encouraged people on social media to engage with nature in their private gardens during lockdown [105]. Other measures employed through conservation campaigns during lockdown included citizen science elements, such as garden bird surveys [106], which added further opportunities for *wildlife-friendly activities*. Engagement with such campaigns (“*@BBCSpringwatch Nice images. We have been watching them building in the garden. I particularly like the way they drop leaves in a ‘nothing to see here’ fashion when other birds get close”)* may have triggered additional interest in wildlife-friendly activities and offer scientific literacy, which is generally valued for its positive societal outcomes [107,108].

There was a strong increase in communicating benefits for mental well-being associated with *wildlife-friendly activities* during Covid-19 lockdown, primarily indicated by people having reported *mitigation of restlessness* and *thankfulness*. *Mitigation of restlessness* included conservation and naturalist activities in the garden (“*Struggling today with being stuck at home, too windy to ring in the garden this morning so sorted out my books. Me lady says I must stop buying bird books... hmmm”)*. Descriptions of *thankfulness* included people feeling fortunate to have a garden in which their children can watch and learn about wildlife (“*Loving these miniature worlds. Just bought my kids a mini-microscope to see what miracles they can be awe-inspired by...we are lucky enough to have a garden, yeast and pond water... #STEM #homeschool #Nature”)*. Increased satisfaction through caring for other living beings could, in part, explain such findings [109], and activities in green spaces more generally can provide benefits for people´s mental well-being through raising nature connectedness [110,111]. People also reported experiencing the benefit for mental well-being of *uplifts* (“*#BlueTit Spotting in our Mum’s Garden. #Nature keeping us sane”)*, which, in part, is driven by biodiverse, wildlife-friendly gardens’ capacity to bolster positive psychological benefits [40]. The mental well-being benefit of *providing hope* was also communicated, rooted in the observation of natural phenomena in the private garden (“*Goldcrest sitting and singing a metre away from us in our garden this morning. Things like this lift the heart in difficult times.”)* and triggering positive future perspectives, a key characteristic of hope [81].

### Wanting a garden

The private garden use category *wanting a garden* appeared nearly exclusively during the 2020 lockdown period. Although not as frequent a theme (relative share of 5%) as the three discussed above, it made distinctly clear the relevance of private gardens during this period of social crisis. Residents without a garden actively expressed the longing for one (“*I sure miss having a garden.*”), or imagined what a difference having one would make during lockdown (“*Having a garden must be so good in times like this”)*, and included comparisons to the social environment (“*My colleague just mentioned she had a socially distanced wine and cheese session in her friend’s front garden and I am SO jealous.*”). These sentiments highlight the value that people appeared to place on being able to access a garden during lockdown (“*I don’t have a garden. I live on the 2nd & 3rd floors […] Missing out on garden time is sad.*”). Not having access to a private garden during lockdown obstructed opportunities to participate in garden activities and gain the associated benefits for mental well-being, which was also widely publicised in the media [112].

Some self-reports indicated that residents might not require a private garden once public venues reopened (“*I am simply enraptured by the thought of having a garden right now. When the bars, restaurants, galleries & theatres open again, I’ll be fine again I’m sure.*”). However, the persistent reports from estate agents of an increase in requests for properties with gardens, patios and balconies since the first Covid-19 lockdown [113] suggest that the desire for access to a private garden remains an important factor for homeowners and tenants.

### DIY (Do-It-Yourself)

People referred to using their garden for *DIY* with a noticeable relative increase (1.4 percentage points) – and a strong increase in absolute terms – during the Covid-19 lockdown. To some, *DIY* might be a key activity in their private garden, but other residents mentioned engaging in *DIY* in their private garden in passing, in a sequence of different events (“*Been for a run, painted the garden fence, now time for Netflix […]*”), making it one more activity that they could turn to during their day. The primary associated benefit for mental well-being identified was *mitigation of restlessness* (“*[…] Day 4: Today’s handyman task involved painting the back garden shed! […]”)*. *DIY* activities, such as woodworking, are associated with positive mood, emotional arousal through making something one needs and enjoyment of the process of making [114].

### Home-based work

Our study shows that the private garden was represented as an extension of the home office during the first Covid-19 lockdown period, with a strong absolute increase therein from 2019 to during lockdown and a slight relative increase (0.7 percentage points). People reported creating make-shift offices in the garden (“*Lockdown office has moved to the garden today... [image shows a man sitting at a simple garden desk, inside a lush garden in very sunny weather]*”). The increase in mentions of *home-based work* as a private garden use during the Covid-19 crisis was, unsurprisingly, driven mainly by the mandated change in work location from the office to people’s homes [115].

Benefits for mental well-being reported for *home-based work* – mentioned more often during lockdown than in the year before – were all been described as *thankfulness*, achieved by working in the private garden or overlooking it (“*For me in London it’s just so wonderful to have the garden door open as I work, and hear no traffic just birdsong*”). Indeed, exposure to natural elements at work is known to improve mental well-being and help manage stress [116].

Some residents expressed benefits from being able to move seamlessly between work and leisure activities in the private garden (“*Between web sales coming in I’ve been […] gardening! […]*”). The private garden was also portrayed as a place of respite from *home-based work* (“*I had a quick walk around my garden during my lunch break today and just enjoyed the sunshine.*‘). Spending work breaks looking at or being in green spaces to relax can be effective in restoring attention at work [117,118], emphasising the value of intersecting private garden use with *home-based work*. These crisis-related changes enhanced the longer-term increase in popularity of *home-based work* [119]. The interest in home-based working in garden environments appears to have persisted beyond the end of the Covid-19 crisis, as indicated by property buyers requesting outdoor spaces that provide shelter, such as garden sheds [120].

### Limitations and planning considerations for future private gardens

Working from social media posts means that we have not obtained direct evidence of garden uses or mental well-being benefits of private gardens but instead determined the discourse around it on the focal platform. Yet, we expect this discourse to connect to realities experienced by those writing these social media posts. To determine how representative our sample is of wider society’s views, in this area and beyond, would require triangulation with different methods. With this study being based on self-reports from a micro-blogging platform, the private garden uses and mental well-being benefits identified will likely not reflect all strata of society equally, for example because households with a higher average age may not use micro-blogging platforms as much as younger ones. Still, the age bias for Twitter data is less in urban populations [121], which likely increases the applicability of our findings to other highly-urbanised areas, particularly in the UK. Individuals in our sample could also be of above-average socio-economic status because having access to a private garden can be a luxury, and designing a garden that provides a variety of opportunities to engage with can be costly, notably when creating high structural diversity through inclusion of trees and large shrubs [122]. Yet, we do not expect this to be a strong effect, given that only about one in five households in Greater London do not have access to a private or shared garden [47] and that many gardens in this area are relatively small [47]. In addition, those people in our sample tweeting about wanting a garden may be of below average socio-economic status (e.g. ground floor flats with private garden are on average 40% more expensive [123]).

Our study showed how private gardens became an important point of conversation during a social crisis. It remains to be determined how far the increased interest in private gardens might persist beyond the end of the Covid-19 crisis, and whether the widened range of private garden uses, including the increased shares of *socialising and leisure activities* and *home-based work*, remains relevant for residents. Furthermore, new social crises will likely continue to emerge, such as the recent increase in energy and food costs associated with the conflict in Ukraine [124], which have the potential to enhance psychological stress. It will thus be important to encourage residents and planners to invest in the potential of private gardens to deliver benefits for mental well-being for different users. Such benefits might be optimised further when the garden displays high biodiversity [125], as people appear more willing to take care of wildlife around them, contributing to higher biodiversity [126] and enhancing connectedness to nature [127]. Open areas for socialising, including lawns, will remain important, suggesting that a mixed design is likely to strengthen the benefits of private gardens for both human mental well-being and nature [128].

The theme *wanting a garden*, which was highly abundant during Covid-19 lockdown, suggests that access to private green space is inadequate for many city residents, despite an alleged 79% of households having access, but that is to a private *or* shared garden, and this value is below the national average (which is 88%) [47,48]. This situation can lead to health problems for the elderly and people in lower socio-economic strata, as became increasingly apparent in the Covid-19 crisis [129]. Securing existing private (and public) urban green spaces is a primary option for improving urban green space planning [130]. Legislating for a minimum percentage of vegetation cover would mitigate long-term loss of private green space through developments such as the paving over of front yards for off-street parking [131]. A more ambitious strategy would involve including long-term and multi-purpose designs for private gardens in housing development plans to enable, on a broader scale, the various garden uses and associated benefits we identified [132]. Many trends in urban planning appear to go in the opposite direction. For example, urban densification through consolidation and infill development, alongside the promotion of active transport, often diminish private and public green space [133]. Strategies to secure sufficient private (and public) green space appear to be sparse [134]. Among other issues, densification might intensify socio-economic inequalities as wealthier residents compensate for the lack of a private garden through having a second (greener) home elsewhere [135]. Some of the self-reported interactions with private gardens were directly related to biodiversity (i.e. *wildlife-friendly activities*, which includes watching birds and other wildlife). Urban planners and conservation organisations involved in renaturing cities campaigns [136] should take this into account, to ensure that multifunctional green spaces include consideration of biodiversity.

## Conclusions

We demonstrated that the Covid-19 crisis transformed the way people thought and talked about their private gardens on Twitter, communicating private garden uses in a range of ways to address the social and mental well-being needs of residents. Our findings show how quickly discourse about private garden use can change, shifting from a focus on display and ornamental purposes to re-setting priorities, reconnecting with nature and appreciating the benefits of taking care of oneself and others, as a community. To support healthy and resilient communities, gardeners, urban planners and related practitioners need to consider the role and breadth of private garden uses reported here and introduce policies, incentives and practices that support long-term maintenance and growth of accessible, biodiverse and multi-purpose private greenspaces.

## Supporting information

S1 Fig Timeline of the first national Covid-19 lockdown in the UK, which began on 24 March 2020 and was extended on 16 April for ‘at least’ three weeks. From 11 May restrictions were handled separately for England, Wales, Scotland and Northern Ireland through conditional plans [87,137]. The period with the strongest impact on people during the first Covid-19 lockdown was 24 March to 10 May, during which the strictest lockdown restrictions were in place before being partially loosened. During this time of strict lockdown, restrictions were ushered in, preventing individuals from leaving their homes except for essential shopping, a daily outdoor exercise session, medical needs and commuting to essential jobs [87]. People were prohibited from meeting friends or family members from a different household [87]. Public parks remained open, but many retail outlets had to close [87].
(TIF)

S2 Fig Tweeting activity on the keyword string “garden” (or “gardened” or “gardening”) in March and April in a 20-mile radius around central London. A comparison of the counts for the posts with the term “garden” from 2017 to 2019 was performed, to find out whether 2019 is a normal pre-Covid year, to then be used in comparison to the Covid crisis year 2020. This plot shows that there is considerable variation in the number of garden-related tweets with each of the years, and that garden conversation peaks may fall in different weeks (cw = calendar week). Across all three years, garden-related spring tweet counts were of a similar order of magnitude, justifying our choice of 2019 to represent a typical pre-lockdown year.
(TIF)

S3 Fig Count of tweets related to the term “garden” (or “gardened” or “gardening”) and average maximum temperature per week, for each of the years 2017, 2018, 2019 and 2020. Graphs show that some peaks in tweet count coincide with relatively high spring temperatures (recorded maximum of 22 °C; cw = calendar week). Yet, other peaks occur in the absence of elevated temperatures or vice versa. Hence, the relationship between warm weather and the volume of garden-related tweets is not clear or straightforward.
(TIF)

S4 Table Initial codes derived while subjecting Twitter posts to thematic analysis. (XLSX)

S5 Fig Histogram showing how many users (user frequency) posted a certain number of tweets (post counts per user) in the sample for both periods, the Covid-19 lockdown period (2020) and the control period (2019). (TIF)

S6 Fig Box plots of background Twitter activity in the study area from 1 March to 31 April, grouped per calendar week, in 2017, 2018 and 2019. The analysis for background Twitter activity was performed for years 2017, 2018, 2019 and 2020, to assess whether twitter activity increased or decreased over these years. The Twitter API full-archive count search was performed for these years. The search string was constructed through the following set of most-commonly occurring English stop words: “the”, “be”, “of”, “and”, “in”, “to”, “have”, “it”, “for”, “you”, “he”, “on”, “with”, “do”, “at”, “by”, “from”, “they”. The search parameters for time frame and geo-location were set like in the initial search for the study sample (March to April, the specified 34-km radius London area). While this approach cannot achieve precise results for the purpose, the tweet counts obtained from this approach still serve as an approximate number for the background Twitter activity in the area and the time frame. Values of 2020 (during lockdown) are within the variations of years 2017–2019 (before lockdown). The activity of tweeting seems to gradually decline over the years 2017–2019 but then increase in 2020, which could have been due to the exceptional (isolated) situation of having lockdown mandates in the Covid-19 crisis. Yet, visual comparison indicates that the tweet counts per calendar week (cw) in the study area and study period do not differ very much. We therefore conclude that the changes from 2019 to 2020, which we analyse in this study, are not subject to any major changes in tweeting behaviour.
(TIF)

S7 Data Sample of 600 tweets (300 in each study period) meeting suitability criteria for thematic analysis, with results on mentioned garden uses and benefits for mental well-being. (PDF)
