# Supplementary figures and images for: Private garden uses and associated mental well-being benefits during the first UK Covid-19 lockdown – a social media investigation
Source: PLoS One. 2026 Apr 8;21(4):e0289446. doi: 10.1371/journal.pone.0289446 (PMC13061261; doi:10.1371/journal.pone.0289446)

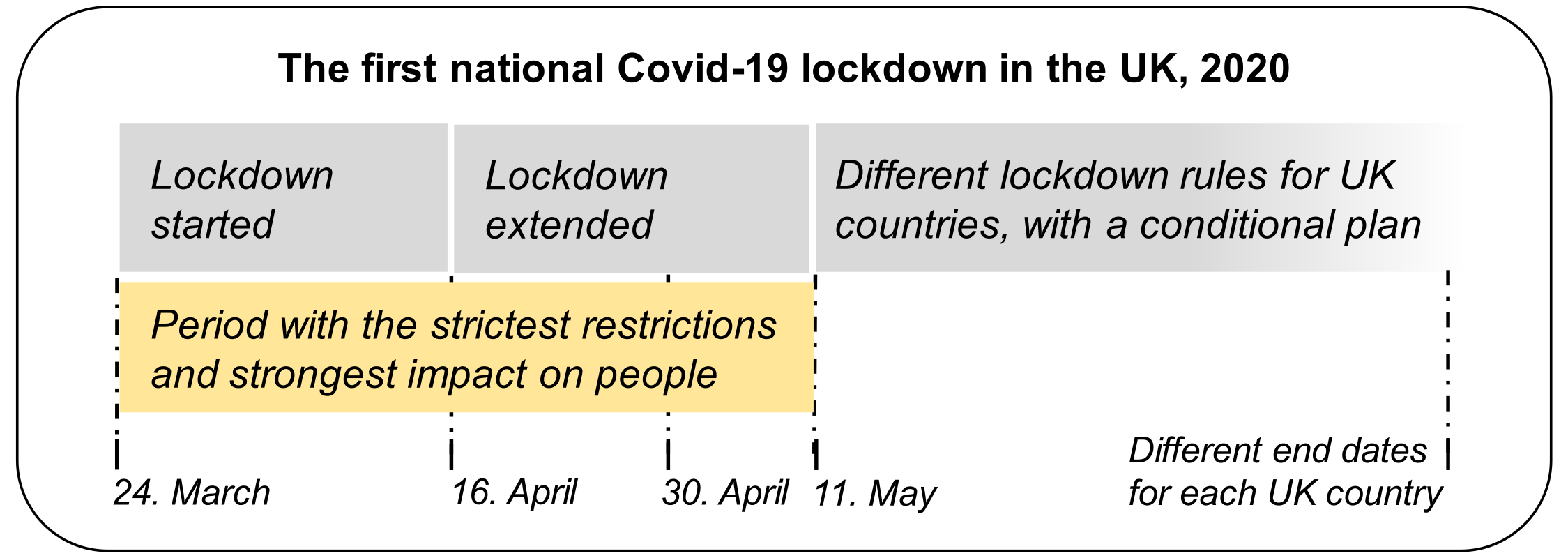

Supplement: S1 Fig — From 11 May restrictions were handled separately for England, Wales, Scotland and Northern Ireland through conditional plans [87,137]. The period with the strongest impact on people during the first Covid-19 lockdown was 24 March to 10 May, during which the strictest lockdown restrictions were in place before being partially loosened. During this time of strict lockdown, restrictions were ushered in, preventing individuals from leaving their homes except for essential shopping, a daily outdoor exercise session, medical needs and commuting to essential jobs [87]. People were prohibited from meeting friends or family members from a different household [87]. Public parks remained open, but many retail outlets had to close [87]. (TIF) [file pone.0289446.s001.tif]

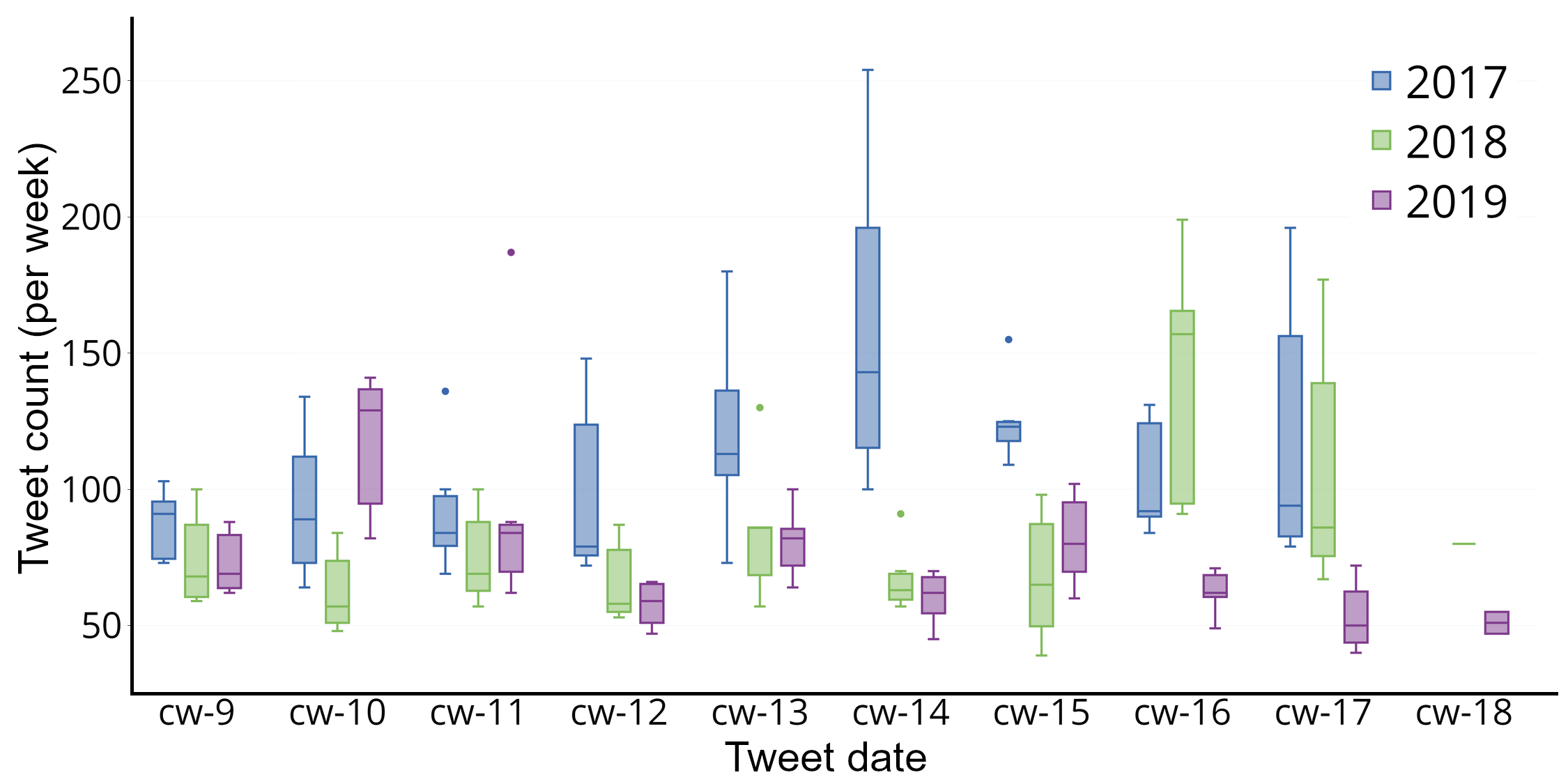

Supplement: S2 Fig — A comparison of the counts for the posts with the term “garden” from 2017 to 2019 was performed, to find out whether 2019 is a normal pre-Covid year, to then be used in comparison to the Covid crisis year 2020. This plot shows that there is considerable variation in the number of garden-related tweets with each of the years, and that garden conversation peaks may fall in different weeks (cw = calendar week). Across all three years, garden-related spring tweet counts were of a similar order of magnitude, justifying our choice of 2019 to represent a typical pre-lockdown year. (TIF) [file pone.0289446.s002.tif]

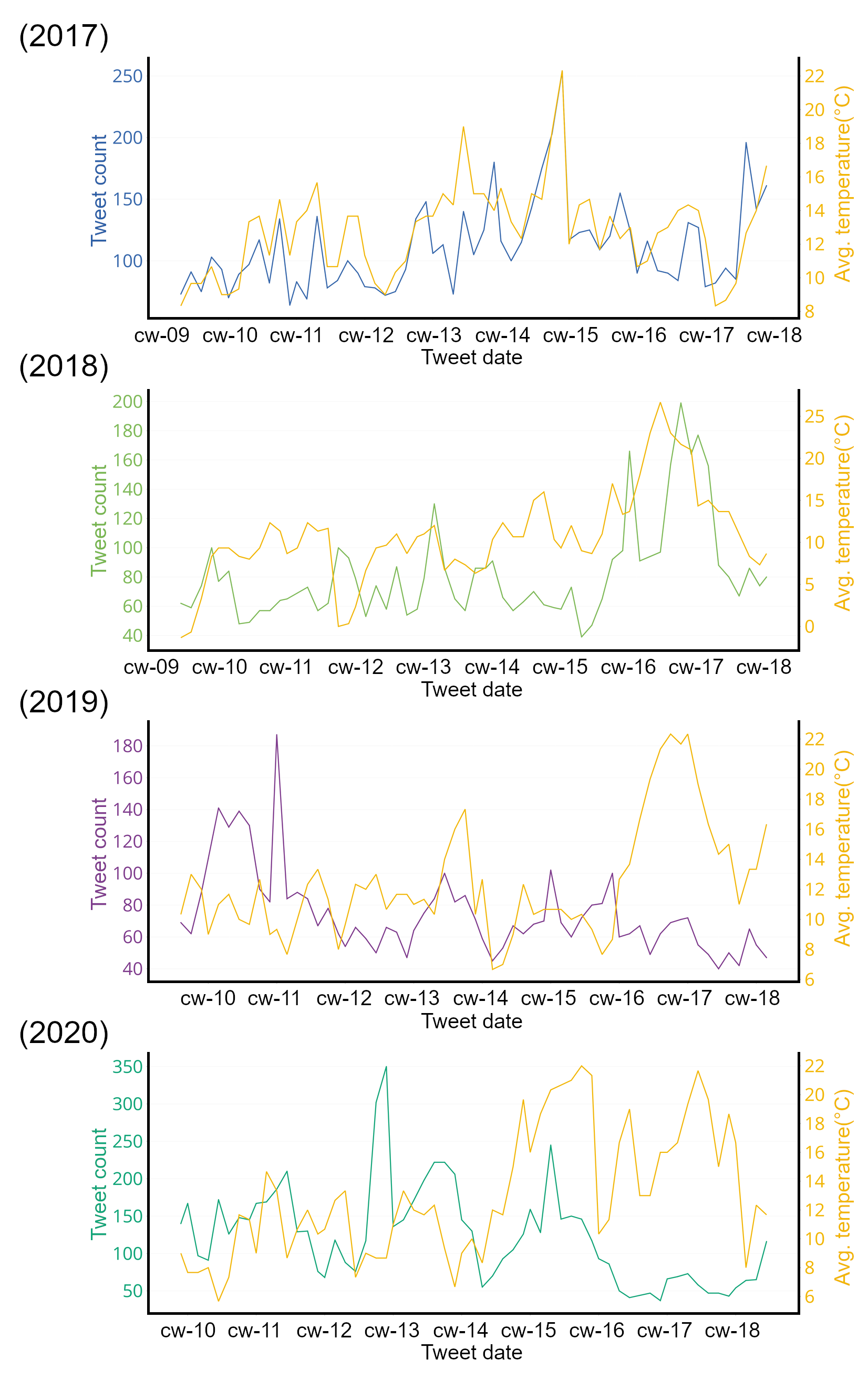

Supplement: S3 Fig — Graphs show that some peaks in tweet count coincide with relatively high spring temperatures (recorded maximum of 22 °C; cw = calendar week). Yet, other peaks occur in the absence of elevated temperatures or vice versa. Hence, the relationship between warm weather and the volume of garden-related tweets is not clear or straightforward. (TIF) [file pone.0289446.s003.tif]

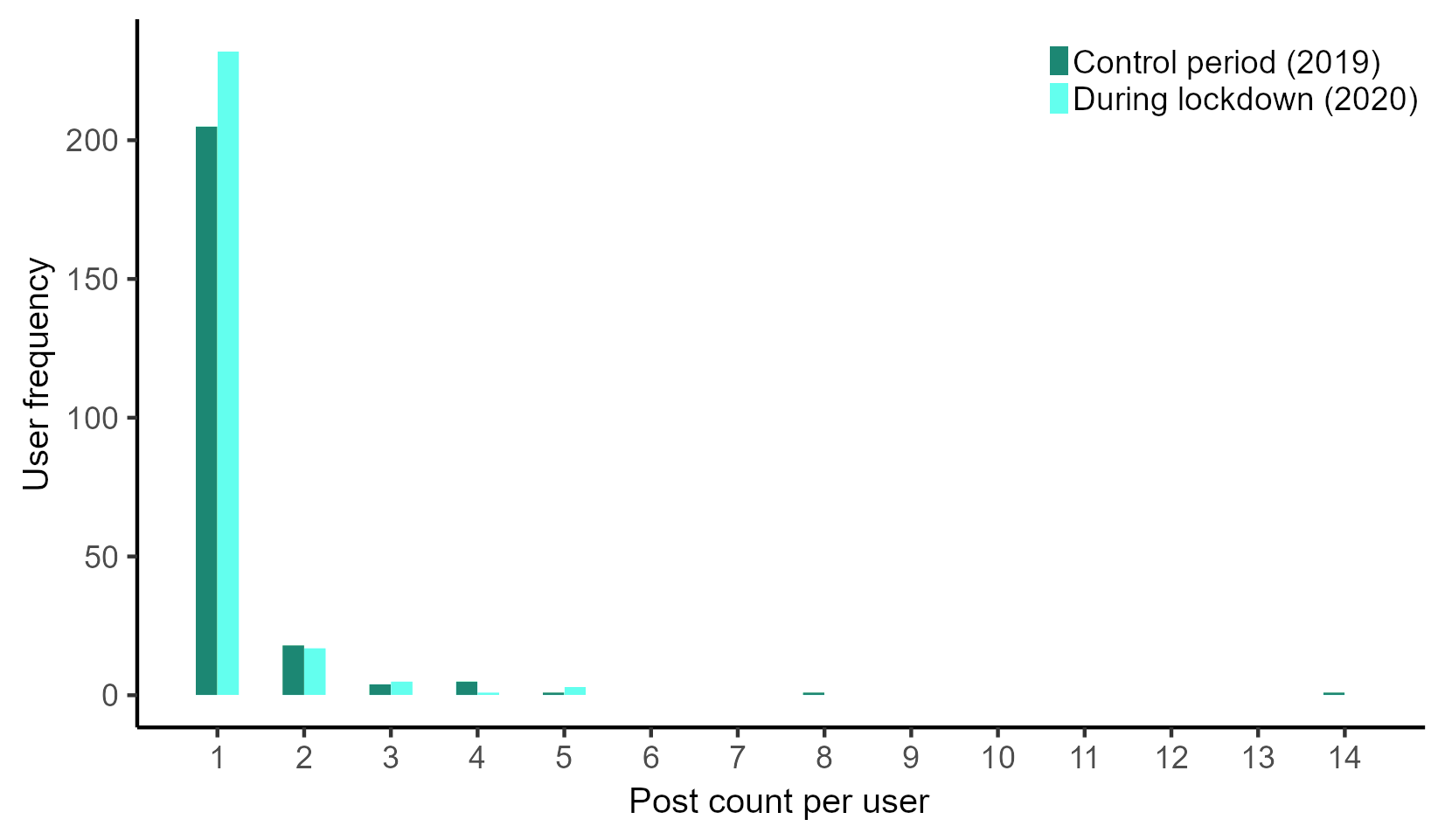

Supplement: S5 Fig — (TIF) [file pone.0289446.s005.tif]

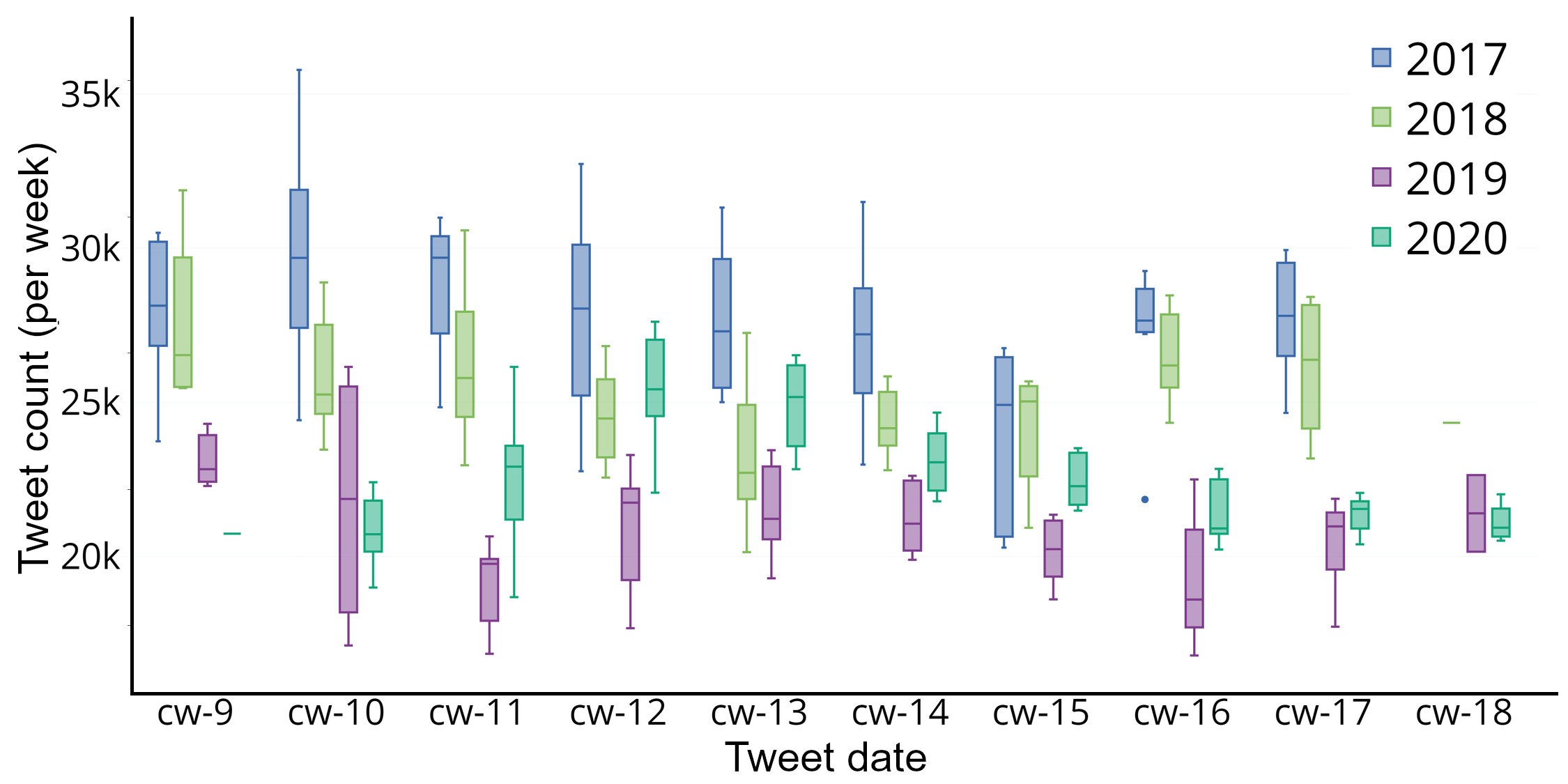

Supplement: S6 Fig — The analysis for background Twitter activity was performed for years 2017, 2018, 2019 and 2020, to assess whether twitter activity increased or decreased over these years. The Twitter API full-archive count search was performed for these years. The search string was constructed through the following set of most-commonly occurring English stop words: “the”, “be”, “of”, “and”, “in”, “to”, “have”, “it”, “for”, “you”, “he”, “on”, “with”, “do”, “at”, “by”, “from”, “they”. The search parameters for time frame and geo-location were set like in the initial search for the study sample (March to April, the specified 34-km radius London area). While this approach cannot achieve precise results for the purpose, the tweet counts obtained from this approach still serve as an approximate number for the background Twitter activity in the area and the time frame. Values of 2020 (during lockdown) are within the variations of years 2017–2019 (before lockdown). The activity of tweeting seems to gradually decline over the years 2017–2019 but then increase in 2020, which could have been due to the exceptional (isolated) situation of having lockdown mandates in the Covid-19 crisis. Yet, visual comparison indicates that the tweet counts per calendar week (cw) in the study area and study period do not differ very much. We therefore conclude that the changes from 2019 to 2020, which we analyse in this study, are not subject to any major changes in tweeting behaviour. (TIF) [file pone.0289446.s006.tif]
