# Supplementary material for: Private garden uses and associated mental well-being benefits during the first UK Covid-19 lockdown – a social media investigation
Source: PLoS One. 2026 Apr 8;21(4):e0289446. doi: 10.1371/journal.pone.0289446 (PMC13061261; doi:10.1371/journal.pone.0289446)
Supplement: S7 Data — (PDF) [file pone.0289446.s007.pdf]

S7 Dataset. Sample of 600 tweets (300 in each study period) meeting suitability criteria for thematic analysis, with results on mentioned garden use and mental well-being benefits.

Tweet id	Study period	Result - garden use	Result – mental well-being benefit
1109748998678761472	2019 (24 Mar - 30 Apr)	socialising and leisure activities	
1109775159760748550	2019 (24 Mar - 30 Apr)	socialising and leisure activities	
1109776736277970949	2019 (24 Mar - 30 Apr)	ornamental and vegetable gardening	
1109788106092412928	2019 (24 Mar - 30 Apr)	socialising and leisure activities	
1109789459829219328	2019 (24 Mar - 30 Apr)	socialising and leisure activities	
1109792362337845248	2019 (24 Mar - 30 Apr)	ornamental and vegetable gardening	
1109811843126779906	2019 (24 Mar - 30 Apr)	socialising and leisure activities	
1109812508783792131	2019 (24 Mar - 30 Apr)	socialising and leisure activities	
1109813423364296706	2019 (24 Mar - 30 Apr)	socialising and leisure activities	
1109813798255378433	2019 (24 Mar - 30 Apr)	socialising and leisure activities	
1109816802501165056	2019 (24 Mar - 30 Apr)	ornamental and vegetable gardening	
1109820536069214210	2019 (24 Mar - 30 Apr)	socialising and leisure activities	thankfulness
1109820566393876480	2019 (24 Mar - 30 Apr)	ornamental and vegetable gardening	
1109827511746203648	2019 (24 Mar - 30 Apr)	socialising and leisure activities	
1109830509666992128	2019 (24 Mar - 30 Apr)	socialising and leisure activities	
1109850288012382210	2019 (24 Mar - 30 Apr)	ornamental and vegetable gardening	
1109855275169792009	2019 (24 Mar - 30 Apr)	socialising and leisure activities	
1109856069365452800	2019 (24 Mar - 30 Apr)	socialising and leisure activities	
1109864686412009472	2019 (24 Mar - 30 Apr)	ornamental and vegetable gardening	
1109865077409308673	2019 (24 Mar - 30 Apr)	ornamental and vegetable gardening	
1109875898990383104	2019 (24 Mar - 30 Apr)	socialising and leisure activities	
1109938533400281089	2019 (24 Mar - 30 Apr)	socialising and leisure activities	thankfulness
1110074311791005698	2019 (24 Mar - 30 Apr)	ornamental and vegetable gardening	

1110076788695289856	2019 (24 Mar - 30 Apr)	socialising and leisure activities	
1110238684966731777	2019 (24 Mar - 30 Apr)	socialising and leisure activities	
1110274846074855426	2019 (24 Mar - 30 Apr)	wildlife-friendly activities	
1110303665276010496	2019 (24 Mar - 30 Apr)	socialising and leisure activities	
1110436815188692994	2019 (24 Mar - 30 Apr)	ornamental and vegetable gardening	
1110456194420916224	2019 (24 Mar - 30 Apr)	ornamental and vegetable gardening	
1110491739687739393	2019 (24 Mar - 30 Apr)	wildlife-friendly activities	
1110522342306996227	2019 (24 Mar - 30 Apr)	socialising and leisure activities	
1110868593934254081	2019 (24 Mar - 30 Apr)	socialising and leisure activities	
1110893034013638656	2019 (24 Mar - 30 Apr)	ornamental and vegetable gardening	
1110905827769102336	2019 (24 Mar - 30 Apr)	wildlife-friendly activities	
1110919065864978436	2019 (24 Mar - 30 Apr)	ornamental and vegetable gardening	
1110921499983134722	2019 (24 Mar - 30 Apr)	wildlife-friendly activities	
1110929046639132674	2019 (24 Mar - 30 Apr)	DIY	
1111215171505188864	2019 (24 Mar - 30 Apr)	ornamental and vegetable gardening	
1111221434481868800	2019 (24 Mar - 30 Apr)	home-based work	
1111233460029607937	2019 (24 Mar - 30 Apr)	socialising and leisure activities	
1111248706932801541	2019 (24 Mar - 30 Apr)	ornamental and vegetable gardening	
1111279182145740800	2019 (24 Mar - 30 Apr)	socialising and leisure activities	
1111291860788674560	2019 (24 Mar - 30 Apr)	socialising and leisure activities	
1111314398075908096	2019 (24 Mar - 30 Apr)	wildlife-friendly activities	
1111314509090770944	2019 (24 Mar - 30 Apr)	wildlife-friendly activities	
1111314891296698369	2019 (24 Mar - 30 Apr)	wildlife-friendly activities	
1111315398262177797	2019 (24 Mar - 30 Apr)	wildlife-friendly activities	
1111335736744333312	2019 (24 Mar - 30 Apr)	ornamental and vegetable gardening	
1111463864292597761	2019 (24 Mar - 30 Apr)	wildlife-friendly activities	
1111526482189402112	2019 (24 Mar - 30 Apr)	socialising and leisure activities	
1111529758234427398	2019 (24 Mar - 30 Apr)	ornamental and vegetable gardening	

1111530977799651328	2019 (24 Mar - 30 Apr)	wildlife-friendly activities	
1111593605892845568	2019 (24 Mar - 30 Apr)	ornamental and vegetable gardening	
1111613753710313472	2019 (24 Mar - 30 Apr)	home-based work	
1111637793514708994	2019 (24 Mar - 30 Apr)	ornamental and vegetable gardening	
1111638580760334338	2019 (24 Mar - 30 Apr)	socialising and leisure activities	
1111642210653622272	2019 (24 Mar - 30 Apr)	socialising and leisure activities	
1111645560312729601	2019 (24 Mar - 30 Apr)	socialising and leisure activities	
1111683400988049408	2019 (24 Mar - 30 Apr)	wildlife-friendly activities	
1111687287002353664	2019 (24 Mar - 30 Apr)	socialising and leisure activities	
1111718972561461248	2019 (24 Mar - 30 Apr)	ornamental and vegetable gardening	
1111883983787311104	2019 (24 Mar - 30 Apr)	socialising and leisure activities	
1111889880978767872	2019 (24 Mar - 30 Apr)	wildlife-friendly activities	
1111927125022556160	2019 (24 Mar - 30 Apr)	socialising and leisure activities	
1111931013033279488	2019 (24 Mar - 30 Apr)	ornamental and vegetable gardening	
1111931431654178818	2019 (24 Mar - 30 Apr)	socialising and leisure activities	
1111943523337732096	2019 (24 Mar - 30 Apr)	ornamental and vegetable gardening	
1111948410339368962	2019 (24 Mar - 30 Apr)	ornamental and vegetable gardening	
1111948463615418368	2019 (24 Mar - 30 Apr)	socialising and leisure activities	
1111949223203926017	2019 (24 Mar - 30 Apr)	socialising and leisure activities	
1111975082962927616	2019 (24 Mar - 30 Apr)	wildlife-friendly activities	
1111992862881792006	2019 (24 Mar - 30 Apr)	socialising and leisure activities	
1111994039329607681	2019 (24 Mar - 30 Apr)	ornamental and vegetable gardening	
1111997399747231745	2019 (24 Mar - 30 Apr)	socialising and leisure activities	contemplation
1111998384452325376	2019 (24 Mar - 30 Apr)	socialising and leisure activities	
1111998551972818944	2019 (24 Mar - 30 Apr)	wildlife-friendly activities	uplifts
1112010169733603328	2019 (24 Mar - 30 Apr)	ornamental and vegetable gardening	
1112014978272366592	2019 (24 Mar - 30 Apr)	ornamental and vegetable gardening	

1112028765608124417	2019 (24 Mar - 30 Apr)	socialising and leisure activities	
1112032574224904193	2019 (24 Mar - 30 Apr)	socialising and leisure activities	
1112035475735396359	2019 (24 Mar - 30 Apr)	ornamental and vegetable gardening	
1112036488043208704	2019 (24 Mar - 30 Apr)	ornamental and vegetable gardening	
1112040117852610561	2019 (24 Mar - 30 Apr)	wildlife-friendly activities	
1112061049686102016	2019 (24 Mar - 30 Apr)	socialising and leisure activities	
1112081736027066368	2019 (24 Mar - 30 Apr)	ornamental and vegetable gardening	
1112294949645631488	2019 (24 Mar - 30 Apr)	ornamental and vegetable gardening	
1112347120470802432	2019 (24 Mar - 30 Apr)	ornamental and vegetable gardening	
1112389073132347392	2019 (24 Mar - 30 Apr)	ornamental and vegetable gardening	
1112437800597667841	2019 (24 Mar - 30 Apr)	ornamental and vegetable gardening	
1112659116646952960	2019 (24 Mar - 30 Apr)	socialising and leisure activities	
1112679956478414848	2019 (24 Mar - 30 Apr)	socialising and leisure activities	
1112771704466624513	2019 (24 Mar - 30 Apr)	ornamental and vegetable gardening	
1112780880999866368	2019 (24 Mar - 30 Apr)	socialising and leisure activities	
1112836452323393537	2019 (24 Mar - 30 Apr)	socialising and leisure activities	
1113004097744048128	2019 (24 Mar - 30 Apr)	ornamental and vegetable gardening	
1113140227512651776	2019 (24 Mar - 30 Apr)	wildlife-friendly activities	
1113148064485830657	2019 (24 Mar - 30 Apr)	wildlife-friendly activities	
1113156398769164288	2019 (24 Mar - 30 Apr)	ornamental and vegetable gardening	
1113195976938881025	2019 (24 Mar - 30 Apr)	DIY	
1113341576829460480	2019 (24 Mar - 30 Apr)	socialising and leisure activities	
1113357171734011904	2019 (24 Mar - 30 Apr)	socialising and leisure activities	
1113806407613210624	2019 (24 Mar - 30 Apr)	ornamental and vegetable gardening	
1113847762716626944	2019 (24 Mar - 30 Apr)	socialising and leisure activities	contemplation
1114065748920557568	2019 (24 Mar - 30 Apr)	ornamental and vegetable gardening	
1114096312457342976	2019 (24 Mar - 30 Apr)	ornamental and vegetable gardening	

1114106493970919425	2019 (24 Mar - 30 Apr)	ornamental and vegetable gardening	thankfulness
1114112865902977025	2019 (24 Mar - 30 Apr)	ornamental and vegetable gardening	uplifts
1114248381449277441	2019 (24 Mar - 30 Apr)	ornamental and vegetable gardening	
1114286660848627721	2019 (24 Mar - 30 Apr)	wildlife-friendly activities	contemplation
1114428721920974848	2019 (24 Mar - 30 Apr)	wildlife-friendly activities	
1114474302261420035	2019 (24 Mar - 30 Apr)	ornamental and vegetable gardening	
1114496359980253184	2019 (24 Mar - 30 Apr)	ornamental and vegetable gardening	
1114505343193747456	2019 (24 Mar - 30 Apr)	wildlife-friendly activities	
1114509670385946624	2019 (24 Mar - 30 Apr)	wildlife-friendly activities	
1114517411020013568	2019 (24 Mar - 30 Apr)	socialising and leisure activities	
1114538504032346118	2019 (24 Mar - 30 Apr)	ornamental and vegetable gardening	
1114542344353787904	2019 (24 Mar - 30 Apr)	ornamental and vegetable gardening	
1114549905832456192	2019 (24 Mar - 30 Apr)	ornamental and vegetable gardening	
1114773671032315905	2019 (24 Mar - 30 Apr)	ornamental and vegetable gardening	
1114810658258866177	2019 (24 Mar - 30 Apr)	home-based work	
1114891360966811654	2019 (24 Mar - 30 Apr)	ornamental and vegetable gardening	
1114921273031843843	2019 (24 Mar - 30 Apr)	ornamental and vegetable gardening	
1114979655390519298	2019 (24 Mar - 30 Apr)	ornamental and vegetable gardening	
1115025866814365697	2019 (24 Mar - 30 Apr)	ornamental and vegetable gardening	
1115165686156931072	2019 (24 Mar - 30 Apr)	wildlife-friendly activities	
1115323660649291776	2019 (24 Mar - 30 Apr)	socialising and leisure activities	
1115336734537965568	2019 (24 Mar - 30 Apr)	wildlife-friendly activities	
1115387708812201985	2019 (24 Mar - 30 Apr)	wildlife-friendly activities	
1115545949475045381	2019 (24 Mar - 30 Apr)	ornamental and vegetable gardening	
1115703704018145281	2019 (24 Mar - 30 Apr)	DIY	
1115730732046016513	2019 (24 Mar - 30 Apr)	wildlife-friendly activities	
1115897596072034304	2019 (24 Mar - 30 Apr)	ornamental and vegetable gardening	

1115907414077902850	2019 (24 Mar - 30 Apr)	ornamental and vegetable gardening	
1115954146522411008	2019 (24 Mar - 30 Apr)	socialising and leisure activities	
1115988349515378688	2019 (24 Mar - 30 Apr)	ornamental and vegetable gardening	
1116000007088480256	2019 (24 Mar - 30 Apr)	ornamental and vegetable gardening	
1116013261424615424	2019 (24 Mar - 30 Apr)	wildlife-friendly activities	
1116014479261696002	2019 (24 Mar - 30 Apr)	ornamental and vegetable gardening	
1116025183331135488	2019 (24 Mar - 30 Apr)	socialising and leisure activities	
1116030919675207680	2019 (24 Mar - 30 Apr)	wildlife-friendly activities	
1116040095117737984	2019 (24 Mar - 30 Apr)	wildlife-friendly activities	
1116073205683425281	2019 (24 Mar - 30 Apr)	ornamental and vegetable gardening	
1116247080006295552	2019 (24 Mar - 30 Apr)	socialising and leisure activities	
1116249933856890880	2019 (24 Mar - 30 Apr)	wildlife-friendly activities	
1116252129163907072	2019 (24 Mar - 30 Apr)	socialising and leisure activities	
1116253777806360576	2019 (24 Mar - 30 Apr)	socialising and leisure activities	
1116328483368710145	2019 (24 Mar - 30 Apr)	DIY	
1116328637823954946	2019 (24 Mar - 30 Apr)	ornamental and vegetable gardening	
1116429264084176896	2019 (24 Mar - 30 Apr)	socialising and leisure activities	
1116574121242976256	2019 (24 Mar - 30 Apr)	socialising and leisure activities	
1116660722380025857	2019 (24 Mar - 30 Apr)	socialising and leisure activities	
1116961434506686464	2019 (24 Mar - 30 Apr)	ornamental and vegetable gardening	
1116961604715913216	2019 (24 Mar - 30 Apr)	wildlife-friendly activities	
1116988885723172864	2019 (24 Mar - 30 Apr)	socialising and leisure activities	
1117000643720425477	2019 (24 Mar - 30 Apr)	socialising and leisure activities	
1117044678300323845	2019 (24 Mar - 30 Apr)	ornamental and vegetable gardening	
1117045937443155969	2019 (24 Mar - 30 Apr)	socialising and leisure activities	
1117053764949749761	2019 (24 Mar - 30 Apr)	socialising and leisure activities	
1117062605598646274	2019 (24 Mar - 30 Apr)	ornamental and vegetable gardening	

1117070698730672129	2019 (24 Mar - 30 Apr)	wildlife-friendly activities	
1117089066347040768	2019 (24 Mar - 30 Apr)	socialising and leisure activities	
1117136022192545792	2019 (24 Mar - 30 Apr)	wildlife-friendly activities	
1117193098449309696	2019 (24 Mar - 30 Apr)	socialising and leisure activities	
1117358556347600897	2019 (24 Mar - 30 Apr)	ornamental and vegetable gardening	
1117410878968803329	2019 (24 Mar - 30 Apr)	home-based work	
1117421121316556802	2019 (24 Mar - 30 Apr)	socialising and leisure activities	
1117423427441315840	2019 (24 Mar - 30 Apr)	socialising and leisure activities	
1117471515203780608	2019 (24 Mar - 30 Apr)	wildlife-friendly activities	
1117495288577646594	2019 (24 Mar - 30 Apr)	wildlife-friendly activities	
1117659773527363585	2019 (24 Mar - 30 Apr)	ornamental and vegetable gardening	
1117711560863096832	2019 (24 Mar - 30 Apr)	wildlife-friendly activities	
1118054420930080768	2019 (24 Mar - 30 Apr)	wildlife-friendly activities	
1118105204208099328	2019 (24 Mar - 30 Apr)	socialising and leisure activities	
1118106395587633152	2019 (24 Mar - 30 Apr)	socialising and leisure activities	
1118124028324134914	2019 (24 Mar - 30 Apr)	socialising and leisure activities	
1118130130700918784	2019 (24 Mar - 30 Apr)	ornamental and vegetable gardening	
1118152977850568704	2019 (24 Mar - 30 Apr)	home-based work	
1118224685118951424	2019 (24 Mar - 30 Apr)	ornamental and vegetable gardening	
1118389137252519940	2019 (24 Mar - 30 Apr)	wildlife-friendly activities	
1118417765881253888	2019 (24 Mar - 30 Apr)	DIY	
1118437162406465536	2019 (24 Mar - 30 Apr)	ornamental and vegetable gardening	uplifts
1118463609116475392	2019 (24 Mar - 30 Apr)	ornamental and vegetable gardening	
1118489416559783936	2019 (24 Mar - 30 Apr)	socialising and leisure activities	
1118521641624064000	2019 (24 Mar - 30 Apr)	socialising and leisure activities	
1118540380067237893	2019 (24 Mar - 30 Apr)	wildlife-friendly activities	
1118550051566051334	2019 (24 Mar - 30 Apr)	ornamental and vegetable gardening	

1118562384128487424	2019 (24 Mar - 30 Apr)	socialising and leisure activities	
1118589806890704896	2019 (24 Mar - 30 Apr)	socialising and leisure activities	
1118604867751170048	2019 (24 Mar - 30 Apr)	wanting a garden	
1118771064274866176	2019 (24 Mar - 30 Apr)	ornamental and vegetable gardening	uplifts
1118773985624363009	2019 (24 Mar - 30 Apr)	wildlife-friendly activities	
1118777167662346241	2019 (24 Mar - 30 Apr)	ornamental and vegetable gardening	
1118838088028102657	2019 (24 Mar - 30 Apr)	ornamental and vegetable gardening	
1118846085089435649	2019 (24 Mar - 30 Apr)	wildlife-friendly activities	
1118846748644896768	2019 (24 Mar - 30 Apr)	ornamental and vegetable gardening	
1118863566067720192	2019 (24 Mar - 30 Apr)	socialising and leisure activities	
1118869029354127360	2019 (24 Mar - 30 Apr)	wildlife-friendly activities	
1118871321293787136	2019 (24 Mar - 30 Apr)	ornamental and vegetable gardening	
1118871500398977024	2019 (24 Mar - 30 Apr)	ornamental and vegetable gardening	
1118898125391958016	2019 (24 Mar - 30 Apr)	socialising and leisure activities	
1118910391407525888	2019 (24 Mar - 30 Apr)	ornamental and vegetable gardening	
1118923902594498562	2019 (24 Mar - 30 Apr)	ornamental and vegetable gardening	
1118937001523392512	2019 (24 Mar - 30 Apr)	DIY	
1118943860103811072	2019 (24 Mar - 30 Apr)	ornamental and vegetable gardening	
1118944459591434240	2019 (24 Mar - 30 Apr)	ornamental and vegetable gardening	
1119134892002922496	2019 (24 Mar - 30 Apr)	socialising and leisure activities	
1119137934668525568	2019 (24 Mar - 30 Apr)	ornamental and vegetable gardening	
1119142734697078784	2019 (24 Mar - 30 Apr)	socialising and leisure activities	
1119162152848244738	2019 (24 Mar - 30 Apr)	ornamental and vegetable gardening	
1119165706459684866	2019 (24 Mar - 30 Apr)	ornamental and vegetable gardening	
1119172046263603200	2019 (24 Mar - 30 Apr)	wildlife-friendly activities	
1119196800710320129	2019 (24 Mar - 30 Apr)	ornamental and vegetable gardening	
1119197129321226240	2019 (24 Mar - 30 Apr)	ornamental and vegetable gardening	

1119203553812332544	2019 (24 Mar - 30 Apr)	socialising and leisure activities	
1119211017353842690	2019 (24 Mar - 30 Apr)	socialising and leisure activities	
1119222233686265856	2019 (24 Mar - 30 Apr)	ornamental and vegetable gardening	
1119228751542390784	2019 (24 Mar - 30 Apr)	socialising and leisure activities	
1119229386350317568	2019 (24 Mar - 30 Apr)	socialising and leisure activities	
1119230970840985600	2019 (24 Mar - 30 Apr)	socialising and leisure activities	
1119233124414578688	2019 (24 Mar - 30 Apr)	socialising and leisure activities	
1119235147801534465	2019 (24 Mar - 30 Apr)	ornamental and vegetable gardening	
1119236570526232576	2019 (24 Mar - 30 Apr)	ornamental and vegetable gardening	
1119246817760247808	2019 (24 Mar - 30 Apr)	socialising and leisure activities	
1119248569754689537	2019 (24 Mar - 30 Apr)	socialising and leisure activities	
1119254649314271235	2019 (24 Mar - 30 Apr)	wildlife-friendly activities	
1119256908852940801	2019 (24 Mar - 30 Apr)	socialising and leisure activities	
1119257247517958144	2019 (24 Mar - 30 Apr)	ornamental and vegetable gardening	
1119260515623219200	2019 (24 Mar - 30 Apr)	socialising and leisure activities	
1119266683301060608	2019 (24 Mar - 30 Apr)	socialising and leisure activities	
1119270961164029952	2019 (24 Mar - 30 Apr)	ornamental and vegetable gardening	
1119271055531610113	2019 (24 Mar - 30 Apr)	socialising and leisure activities	
1119277380575436802	2019 (24 Mar - 30 Apr)	ornamental and vegetable gardening	
1119279537513218049	2019 (24 Mar - 30 Apr)	ornamental and vegetable gardening	
1119293451911086080	2019 (24 Mar - 30 Apr)	ornamental and vegetable gardening	
1119323232396558336	2019 (24 Mar - 30 Apr)	ornamental and vegetable gardening	
1119325814691192833	2019 (24 Mar - 30 Apr)	ornamental and vegetable gardening	
1119328146610884609	2019 (24 Mar - 30 Apr)	ornamental and vegetable gardening	
1119344643693395968	2019 (24 Mar - 30 Apr)	DIY	
1119344759653437441	2019 (24 Mar - 30 Apr)	socialising and leisure activities	
1119344873004568577	2019 (24 Mar - 30 Apr)	wildlife-friendly activities	

1119352011085045762	2019 (24 Mar - 30 Apr)	ornamental and vegetable gardening	
1119384297838977024	2019 (24 Mar - 30 Apr)	socialising and leisure activities	
1119465162094682113	2019 (24 Mar - 30 Apr)	ornamental and vegetable gardening	
1119486121652707329	2019 (24 Mar - 30 Apr)	socialising and leisure activities	
1119568690498945024	2019 (24 Mar - 30 Apr)	socialising and leisure activities	
1119575430284156928	2019 (24 Mar - 30 Apr)	ornamental and vegetable gardening	
1119576342209662977	2019 (24 Mar - 30 Apr)	ornamental and vegetable gardening	
1119582509564661760	2019 (24 Mar - 30 Apr)	ornamental and vegetable gardening	
1120016763834576898	2019 (24 Mar - 30 Apr)	socialising and leisure activities	
1120038076686925824	2019 (24 Mar - 30 Apr)	socialising and leisure activities	
1120049907484844037	2019 (24 Mar - 30 Apr)	wildlife-friendly activities	
1120060640318586880	2019 (24 Mar - 30 Apr)	socialising and leisure activities	
1121382893891280896	2019 (24 Mar - 30 Apr)	ornamental and vegetable gardening	
1121422098956681217	2019 (24 Mar - 30 Apr)	ornamental and vegetable gardening	
1121430739898253312	2019 (24 Mar - 30 Apr)	ornamental and vegetable gardening	
1121434880733216768	2019 (24 Mar - 30 Apr)	ornamental and vegetable gardening	
1121440391134232578	2019 (24 Mar - 30 Apr)	wildlife-friendly activities	
1121454119313657856	2019 (24 Mar - 30 Apr)	ornamental and vegetable gardening	
1121523292941189122	2019 (24 Mar - 30 Apr)	socialising and leisure activities	
1121660275952717824	2019 (24 Mar - 30 Apr)	ornamental and vegetable gardening	
1121660846264897536	2019 (24 Mar - 30 Apr)	wildlife-friendly activities	
1121710063536701440	2019 (24 Mar - 30 Apr)	ornamental and vegetable gardening	
1121759632719060993	2019 (24 Mar - 30 Apr)	socialising and leisure activities	
1121795206267883521	2019 (24 Mar - 30 Apr)	ornamental and vegetable gardening	
1121796446276087810	2019 (24 Mar - 30 Apr)	ornamental and vegetable gardening	
1121839329637089281	2019 (24 Mar - 30 Apr)	socialising and leisure activities	
1121867815701635073	2019 (24 Mar - 30 Apr)	socialising and leisure activities	

1121909030761447424	2019 (24 Mar - 30 Apr)	wildlife-friendly activities	
1122028104057348096	2019 (24 Mar - 30 Apr)	home-based work	
1122053942026784768	2019 (24 Mar - 30 Apr)	ornamental and vegetable gardening	
1122055457139580929	2019 (24 Mar - 30 Apr)	ornamental and vegetable gardening	
1122121507478614017	2019 (24 Mar - 30 Apr)	socialising and leisure activities	
1122123411604553728	2019 (24 Mar - 30 Apr)	ornamental and vegetable gardening	
1122131634327658496	2019 (24 Mar - 30 Apr)	ornamental and vegetable gardening	
1122172473296945153	2019 (24 Mar - 30 Apr)	ornamental and vegetable gardening	
1122194394952675330	2019 (24 Mar - 30 Apr)	ornamental and vegetable gardening	
1122206673152561152	2019 (24 Mar - 30 Apr)	wildlife-friendly activities	
1122209400951848966	2019 (24 Mar - 30 Apr)	ornamental and vegetable gardening	
1122409023691685888	2019 (24 Mar - 30 Apr)	socialising and leisure activities	
1122422495653416960	2019 (24 Mar - 30 Apr)	socialising and leisure activities	
1122473611300728838	2019 (24 Mar - 30 Apr)	ornamental and vegetable gardening	
1122492846311989248	2019 (24 Mar - 30 Apr)	ornamental and vegetable gardening	
1122516364315508736	2019 (24 Mar - 30 Apr)	ornamental and vegetable gardening	
1122523828025004034	2019 (24 Mar - 30 Apr)	wildlife-friendly activities	
1122529755830603777	2019 (24 Mar - 30 Apr)	wildlife-friendly activities	
1122545916794241029	2019 (24 Mar - 30 Apr)	wildlife-friendly activities	
1122551547781685248	2019 (24 Mar - 30 Apr)	wildlife-friendly activities	
1122568674626482187	2019 (24 Mar - 30 Apr)	wildlife-friendly activities	
1122569731507138562	2019 (24 Mar - 30 Apr)	socialising and leisure activities	
1122571209688997890	2019 (24 Mar - 30 Apr)	wildlife-friendly activities	
1122588165825548288	2019 (24 Mar - 30 Apr)	ornamental and vegetable gardening	
1122798268239568896	2019 (24 Mar - 30 Apr)	socialising and leisure activities	
1122857272437293061	2019 (24 Mar - 30 Apr)	ornamental and vegetable gardening	
1122876566466367489	2019 (24 Mar - 30 Apr)	DIY	

1122884560918929411	2019 (24 Mar - 30 Apr)	DIY	
1122906337745166336	2019 (24 Mar - 30 Apr)	DIY	
1122913911978827777	2019 (24 Mar - 30 Apr)	ornamental and vegetable gardening	
1122914235472842752	2019 (24 Mar - 30 Apr)	wildlife-friendly activities	
1122951615051386880	2019 (24 Mar - 30 Apr)	ornamental and vegetable gardening	
1123013607665688577	2019 (24 Mar - 30 Apr)	wildlife-friendly activities	
1243292879776296960	2020 (24 Mar - 30 Apr)	socialising and leisure activities	
1243297650377703429	2020 (24 Mar - 30 Apr)	wanting a garden	
1243301857151377408	2020 (24 Mar - 30 Apr)	ornamental and vegetable gardening	mitigation of restlessness
1243307844792975362	2020 (24 Mar - 30 Apr)	socialising and leisure activities	
1243318859937075200	2020 (24 Mar - 30 Apr)	ornamental and vegetable gardening	
1243402127117922304	2020 (24 Mar - 30 Apr)	home-based work	
1243426323340464129	2020 (24 Mar - 30 Apr)	socialising and leisure activities	mitigation of restlessness
1243428464667103232	2020 (24 Mar - 30 Apr)	socialising and leisure activities	
1243435540793511936	2020 (24 Mar - 30 Apr)	socialising and leisure activities	
1243435722314481664	2020 (24 Mar - 30 Apr)	socialising and leisure activities	
1243445620251267072	2020 (24 Mar - 30 Apr)	socialising and leisure activities	thankfulness
1243449504088698880	2020 (24 Mar - 30 Apr)	wanting a garden	
1243454949230551040	2020 (24 Mar - 30 Apr)	socialising and leisure activities	contemplation
1243455578975977473	2020 (24 Mar - 30 Apr)	wanting a garden	
1243457866310320130	2020 (24 Mar - 30 Apr)	wildlife-friendly activities	
1243458105754750976	2020 (24 Mar - 30 Apr)	wanting a garden	
1243462251048325120	2020 (24 Mar - 30 Apr)	socialising and leisure activities	
1243463780442558464	2020 (24 Mar - 30 Apr)	ornamental and vegetable gardening	mitigation of restlessness
1243463830677729280	2020 (24 Mar - 30 Apr)	socialising and leisure activities	
1243465111408443392	2020 (24 Mar - 30 Apr)	ornamental and vegetable gardening	
1243469704804663310	2020 (24 Mar - 30 Apr)	socialising and leisure activities	
1243470815292805121	2020 (24 Mar - 30 Apr)	wildlife-friendly activities	

1243473977303015425	2020 (24 Mar - 30 Apr)	DIY	
1243489935757398017	2020 (24 Mar - 30 Apr)	socialising and leisure activities	
1243490257116504065	2020 (24 Mar - 30 Apr)	socialising and leisure activities	
1243490393582440448	2020 (24 Mar - 30 Apr)	socialising and leisure activities	
1243492178711773185	2020 (24 Mar - 30 Apr)	socialising and leisure activities	mitigation of restlessness
1243492740278759425	2020 (24 Mar - 30 Apr)	ornamental and vegetable gardening	
1243497728467968003	2020 (24 Mar - 30 Apr)	socialising and leisure activities	
1243514615260155909	2020 (24 Mar - 30 Apr)	ornamental and vegetable gardening	
1243523789264228353	2020 (24 Mar - 30 Apr)	socialising and leisure activities	
1243524948666118144	2020 (24 Mar - 30 Apr)	ornamental and vegetable gardening	
1243529458855940096	2020 (24 Mar - 30 Apr)	ornamental and vegetable gardening	
1243533201169166336	2020 (24 Mar - 30 Apr)	wildlife-friendly activities	
1243542387370188807	2020 (24 Mar - 30 Apr)	socialising and leisure activities	mitigation of restlessness
1243548216425164802	2020 (24 Mar - 30 Apr)	socialising and leisure activities	
1243548562144866307	2020 (24 Mar - 30 Apr)	ornamental and vegetable gardening	
1243552583605772295	2020 (24 Mar - 30 Apr)	ornamental and vegetable gardening	
1243556904468647940	2020 (24 Mar - 30 Apr)	socialising and leisure activities	
1243557803643547649	2020 (24 Mar - 30 Apr)	socialising and leisure activities	
1243560441848176640	2020 (24 Mar - 30 Apr)	ornamental and vegetable gardening	
1243561594761760775	2020 (24 Mar - 30 Apr)	socialising and leisure activities	mitigation of restlessness
1243564613935026177	2020 (24 Mar - 30 Apr)	DIY	mitigation of restlessness
1243579447976083456	2020 (24 Mar - 30 Apr)	wanting a garden	
1243582006723780613	2020 (24 Mar - 30 Apr)	ornamental and vegetable gardening	
1243582915436576768	2020 (24 Mar - 30 Apr)	ornamental and vegetable gardening	
1243584868665233413	2020 (24 Mar - 30 Apr)	ornamental and vegetable gardening	
1243600582104973320	2020 (24 Mar - 30 Apr)	socialising and leisure activities	mitigation of restlessness
1243603429244764160	2020 (24 Mar - 30 Apr)	ornamental and vegetable gardening	

1243604416051261441	2020 (24 Mar - 30 Apr)	wildlife-friendly activities	
1243605827682000896	2020 (24 Mar - 30 Apr)	ornamental and vegetable gardening	
1243607576417370115	2020 (24 Mar - 30 Apr)	ornamental and vegetable gardening	providing hope
1243610537436602374	2020 (24 Mar - 30 Apr)	socialising and leisure activities	thankfulness
1243614026791129089	2020 (24 Mar - 30 Apr)	socialising and leisure activities	thankfulness
1243616475065221120	2020 (24 Mar - 30 Apr)	ornamental and vegetable gardening	
1243617140978192384	2020 (24 Mar - 30 Apr)	home-based work	
1243618626227384321	2020 (24 Mar - 30 Apr)	wildlife-friendly activities	
1243620992246824964	2020 (24 Mar - 30 Apr)	socialising and leisure activities	
1243621578862190592	2020 (24 Mar - 30 Apr)	socialising and leisure activities	providing hope
1243625044321992707	2020 (24 Mar - 30 Apr)	wanting a garden	
1243625347188502533	2020 (24 Mar - 30 Apr)	socialising and leisure activities	mitigation of restlessness
1243639325394337801	2020 (24 Mar - 30 Apr)	ornamental and vegetable gardening	
1243657932014850049	2020 (24 Mar - 30 Apr)	socialising and leisure activities	
1243676765404499970	2020 (24 Mar - 30 Apr)	wanting a garden	
1243707963719716870	2020 (24 Mar - 30 Apr)	socialising and leisure activities	thankfulness
1243786425067352064	2020 (24 Mar - 30 Apr)	ornamental and vegetable gardening	
1243796143223590912	2020 (24 Mar - 30 Apr)	ornamental and vegetable gardening	
1243798687869960192	2020 (24 Mar - 30 Apr)	ornamental and vegetable gardening	
1243799480090202112	2020 (24 Mar - 30 Apr)	wildlife-friendly activities	
1243805363385835522	2020 (24 Mar - 30 Apr)	ornamental and vegetable gardening	
1243813379090853889	2020 (24 Mar - 30 Apr)	ornamental and vegetable gardening	
1243816133544882176	2020 (24 Mar - 30 Apr)	ornamental and vegetable gardening	
1243822705071226886	2020 (24 Mar - 30 Apr)	DIY	mitigation of restlessness
1243824331500765184	2020 (24 Mar - 30 Apr)	ornamental and vegetable gardening	
1243835264302268416	2020 (24 Mar - 30 Apr)	wildlife-friendly activities	
1243844014790631426	2020 (24 Mar - 30 Apr)	wildlife-friendly activities	

1243850614850957312	2020 (24 Mar - 30 Apr)	wanting a garden	
1243852250230984705	2020 (24 Mar - 30 Apr)	wildlife-friendly activities	
1243854913165115392	2020 (24 Mar - 30 Apr)	ornamental and vegetable gardening	thankfulness
1243857640251408386	2020 (24 Mar - 30 Apr)	wanting a garden	
1243859238318936064	2020 (24 Mar - 30 Apr)	socialising and leisure activities	
1243867269882613760	2020 (24 Mar - 30 Apr)	socialising and leisure activities	
1243872259338625024	2020 (24 Mar - 30 Apr)	wildlife-friendly activities	mitigation of restlessness
1243877404193566721	2020 (24 Mar - 30 Apr)	wildlife-friendly activities	
1243877548414697472	2020 (24 Mar - 30 Apr)	socialising and leisure activities	mitigation of restlessness
1243878233885548544	2020 (24 Mar - 30 Apr)	ornamental and vegetable gardening	
1243879601933352962	2020 (24 Mar - 30 Apr)	ornamental and vegetable gardening	
1243879921493082119	2020 (24 Mar - 30 Apr)	ornamental and vegetable gardening	
1243882914661761024	2020 (24 Mar - 30 Apr)	DIY	
1243887141219790849	2020 (24 Mar - 30 Apr)	socialising and leisure activities	mitigation of restlessness
1243888328623247365	2020 (24 Mar - 30 Apr)	ornamental and vegetable gardening	
1243888448261554177	2020 (24 Mar - 30 Apr)	ornamental and vegetable gardening	
1243893224785301504	2020 (24 Mar - 30 Apr)	ornamental and vegetable gardening	
1243904824980144128	2020 (24 Mar - 30 Apr)	socialising and leisure activities	
1243905394931621890	2020 (24 Mar - 30 Apr)	socialising and leisure activities	
1243905961942822912	2020 (24 Mar - 30 Apr)	DIY	
1243908813805273089	2020 (24 Mar - 30 Apr)	DIY	
1243910957316833280	2020 (24 Mar - 30 Apr)	ornamental and vegetable gardening	
1243911443050889216	2020 (24 Mar - 30 Apr)	ornamental and vegetable gardening	
1243919265763332099	2020 (24 Mar - 30 Apr)	socialising and leisure activities	
1243924414640652288	2020 (24 Mar - 30 Apr)	ornamental and vegetable gardening	
1243929288476438529	2020 (24 Mar - 30 Apr)	wildlife-friendly activities	
1243931696921219074	2020 (24 Mar - 30 Apr)	wildlife-friendly activities	
1243940909252849664	2020 (24 Mar - 30 Apr)	wildlife-friendly activities	

1243941697404506112	2020 (24 Mar - 30 Apr)	wildlife-friendly activities	
1243949909105152000	2020 (24 Mar - 30 Apr)	socialising and leisure activities	
1243959575683964928	2020 (24 Mar - 30 Apr)	wildlife-friendly activities	mitigation of restlessness
1243961840243281921	2020 (24 Mar - 30 Apr)	wildlife-friendly activities	
1243967483163430912	2020 (24 Mar - 30 Apr)	socialising and leisure activities	
1243968760945483776	2020 (24 Mar - 30 Apr)	DIY	
1243991058469568512	2020 (24 Mar - 30 Apr)	wildlife-friendly activities	
1244000242925809666	2020 (24 Mar - 30 Apr)	socialising and leisure activities	
1244013951144333312	2020 (24 Mar - 30 Apr)	wildlife-friendly activities	providing hope
1244020289631145986	2020 (24 Mar - 30 Apr)	wildlife-friendly activities	
1244028195801833473	2020 (24 Mar - 30 Apr)	socialising and leisure activities	thankfulness
1244118379344154627	2020 (24 Mar - 30 Apr)	wanting a garden	
1244157158352175104	2020 (24 Mar - 30 Apr)	ornamental and vegetable gardening	
1244158167686295554	2020 (24 Mar - 30 Apr)	DIY	
1244168744198430720	2020 (24 Mar - 30 Apr)	wildlife-friendly activities	
1244185671708934145	2020 (24 Mar - 30 Apr)	DIY	
1244190795034505217	2020 (24 Mar - 30 Apr)	ornamental and vegetable gardening	
1244195517987180544	2020 (24 Mar - 30 Apr)	socialising and leisure activities	mitigation of restlessness
1244203282479882240	2020 (24 Mar - 30 Apr)	ornamental and vegetable gardening	
1244205412632707072	2020 (24 Mar - 30 Apr)	wildlife-friendly activities	
1244208245784186880	2020 (24 Mar - 30 Apr)	ornamental and vegetable gardening	
1244216400400584705	2020 (24 Mar - 30 Apr)	ornamental and vegetable gardening	
1244221178002235392	2020 (24 Mar - 30 Apr)	socialising and leisure activities	
1244226922365612034	2020 (24 Mar - 30 Apr)	wanting a garden	
1244237012498030593	2020 (24 Mar - 30 Apr)	ornamental and vegetable gardening	
1244240974517358592	2020 (24 Mar - 30 Apr)	socialising and leisure activities	
1244250434296037376	2020 (24 Mar - 30 Apr)	socialising and leisure activities	

1244254696228995073	2020 (24 Mar - 30 Apr)	ornamental and vegetable gardening	
1244275364878266369	2020 (24 Mar - 30 Apr)	wanting a garden	
1244278216640118785	2020 (24 Mar - 30 Apr)	socialising and leisure activities	
1244285613152534528	2020 (24 Mar - 30 Apr)	wildlife-friendly activities	
1244286395704905730	2020 (24 Mar - 30 Apr)	wildlife-friendly activities	uplifts
1244304792878530562	2020 (24 Mar - 30 Apr)	wildlife-friendly activities	
1244313338449932288	2020 (24 Mar - 30 Apr)	ornamental and vegetable gardening	
1244317505390182401	2020 (24 Mar - 30 Apr)	wildlife-friendly activities	
1244320257227001859	2020 (24 Mar - 30 Apr)	socialising and leisure activities	
1244323021915066368	2020 (24 Mar - 30 Apr)	socialising and leisure activities	
1244330483938398210	2020 (24 Mar - 30 Apr)	socialising and leisure activities	uplifts
1244338364658171904	2020 (24 Mar - 30 Apr)	wildlife-friendly activities	
1244341558746456066	2020 (24 Mar - 30 Apr)	wildlife-friendly activities	
1244372669044269056	2020 (24 Mar - 30 Apr)	socialising and leisure activities	
1244373226609954816	2020 (24 Mar - 30 Apr)	socialising and leisure activities	
1244383115260428292	2020 (24 Mar - 30 Apr)	ornamental and vegetable gardening	
1244407465531277313	2020 (24 Mar - 30 Apr)	socialising and leisure activities	
1244426484367966209	2020 (24 Mar - 30 Apr)	ornamental and vegetable gardening	mitigation of restlessness
1244540008939257857	2020 (24 Mar - 30 Apr)	DIY	
1244547247393685505	2020 (24 Mar - 30 Apr)	socialising and leisure activities	
1244553309811343360	2020 (24 Mar - 30 Apr)	wildlife-friendly activities	
1244554820213768194	2020 (24 Mar - 30 Apr)	ornamental and vegetable gardening	
1244568521062985729	2020 (24 Mar - 30 Apr)	socialising and leisure activities	
1244569100870959104	2020 (24 Mar - 30 Apr)	wildlife-friendly activities	
1244578032410660865	2020 (24 Mar - 30 Apr)	ornamental and vegetable gardening	
1244585291366703104	2020 (24 Mar - 30 Apr)	wildlife-friendly activities	
1244604751565787139	2020 (24 Mar - 30 Apr)	socialising and leisure activities	

1244622260712988673	2020 (24 Mar - 30 Apr)	ornamental and vegetable gardening	
1244646683880890368	2020 (24 Mar - 30 Apr)	socialising and leisure activities	
1244664109230166021	2020 (24 Mar - 30 Apr)	socialising and leisure activities	uplifts
1244667205997993984	2020 (24 Mar - 30 Apr)	wildlife-friendly activities	
1244667721301852161	2020 (24 Mar - 30 Apr)	socialising and leisure activities	mitigation of restlessness
1244672854844682240	2020 (24 Mar - 30 Apr)	socialising and leisure activities	
1244692790212276225	2020 (24 Mar - 30 Apr)	socialising and leisure activities	
1244698027731963905	2020 (24 Mar - 30 Apr)	socialising and leisure activities	
1244698291541028865	2020 (24 Mar - 30 Apr)	ornamental and vegetable gardening	
1244699554752192516	2020 (24 Mar - 30 Apr)	socialising and leisure activities	uplifts
1244700712891822087	2020 (24 Mar - 30 Apr)	socialising and leisure activities	uplifts
1244701217474973697	2020 (24 Mar - 30 Apr)	wildlife-friendly activities	thankfulness
1244705547619835909	2020 (24 Mar - 30 Apr)	socialising and leisure activities	
1244710901866999812	2020 (24 Mar - 30 Apr)	socialising and leisure activities	
1244718178653147139	2020 (24 Mar - 30 Apr)	socialising and leisure activities	
1244719857779818500	2020 (24 Mar - 30 Apr)	wildlife-friendly activities	
1244741756245225480	2020 (24 Mar - 30 Apr)	DIY	
1244765168137121792	2020 (24 Mar - 30 Apr)	socialising and leisure activities	
1249322419866808320	2020 (24 Mar - 30 Apr)	wildlife-friendly activities	
1249323292323913728	2020 (24 Mar - 30 Apr)	socialising and leisure activities	
1249325786982645761	2020 (24 Mar - 30 Apr)	socialising and leisure activities	
1249327961871994880	2020 (24 Mar - 30 Apr)	socialising and leisure activities	providing hope
1249328025591824384	2020 (24 Mar - 30 Apr)	socialising and leisure activities	
1249334494097739778	2020 (24 Mar - 30 Apr)	socialising and leisure activities	
1249334909434544129	2020 (24 Mar - 30 Apr)	socialising and leisure activities	
1249335059506581512	2020 (24 Mar - 30 Apr)	socialising and leisure activities	
1249585303653888000	2020 (24 Mar - 30 Apr)	socialising and leisure activities	
1249588081461727237	2020 (24 Mar - 30 Apr)	wildlife-friendly activities	

1249591195476668416	2020 (24 Mar - 30 Apr)	ornamental and vegetable gardening	
1249607156279791616	2020 (24 Mar - 30 Apr)	socialising and leisure activities	uplifts
1249608049389711360	2020 (24 Mar - 30 Apr)	ornamental and vegetable gardening	
1249613360016101384	2020 (24 Mar - 30 Apr)	socialising and leisure activities	providing hope
1249614482160128002	2020 (24 Mar - 30 Apr)	wildlife-friendly activities	
1249620862824853505	2020 (24 Mar - 30 Apr)	wildlife-friendly activities	
1249951313632378880	2020 (24 Mar - 30 Apr)	socialising and leisure activities	
1249954220712833025	2020 (24 Mar - 30 Apr)	socialising and leisure activities	
1250010395961671687	2020 (24 Mar - 30 Apr)	socialising and leisure activities	
1250013571804692480	2020 (24 Mar - 30 Apr)	home-based work	
1250076470694862849	2020 (24 Mar - 30 Apr)	socialising and leisure activities	
1250090062919974912	2020 (24 Mar - 30 Apr)	socialising and leisure activities	
1250135324510076929	2020 (24 Mar - 30 Apr)	ornamental and vegetable gardening	
1250137262282747913	2020 (24 Mar - 30 Apr)	ornamental and vegetable gardening	
1250305702540627968	2020 (24 Mar - 30 Apr)	socialising and leisure activities	
1250314377921585153	2020 (24 Mar - 30 Apr)	wildlife-friendly activities	
1250374090873061376	2020 (24 Mar - 30 Apr)	socialising and leisure activities	
1250380348673921024	2020 (24 Mar - 30 Apr)	ornamental and vegetable gardening	
1250428407134871552	2020 (24 Mar - 30 Apr)	socialising and leisure activities	
1250429583247081472	2020 (24 Mar - 30 Apr)	ornamental and vegetable gardening	
1250485709129093128	2020 (24 Mar - 30 Apr)	ornamental and vegetable gardening	uplifts
1250489151109181444	2020 (24 Mar - 30 Apr)	DIY	
1250666756177383424	2020 (24 Mar - 30 Apr)	socialising and leisure activities	
1250726616939913218	2020 (24 Mar - 30 Apr)	ornamental and vegetable gardening	
1250727240456982529	2020 (24 Mar - 30 Apr)	socialising and leisure activities	
1250787798963060736	2020 (24 Mar - 30 Apr)	socialising and leisure activities	
1250803202716504064	2020 (24 Mar - 30 Apr)	ornamental and vegetable gardening	

1250855039633166336	2020 (24 Mar - 30 Apr)	wanting a garden	
1250855779349016576	2020 (24 Mar - 30 Apr)	socialising and leisure activities	
1251034140503412737	2020 (24 Mar - 30 Apr)	ornamental and vegetable gardening	uplifts
1251041184182648832	2020 (24 Mar - 30 Apr)	wildlife-friendly activities	
1251088432279031809	2020 (24 Mar - 30 Apr)	wildlife-friendly activities	
1251096433442328577	2020 (24 Mar - 30 Apr)	wildlife-friendly activities	
1251158946175348736	2020 (24 Mar - 30 Apr)	socialising and leisure activities	
1251159921246105601	2020 (24 Mar - 30 Apr)	socialising and leisure activities	
1251220919256743939	2020 (24 Mar - 30 Apr)	ornamental and vegetable gardening	uplifts
1251225502657937408	2020 (24 Mar - 30 Apr)	ornamental and vegetable gardening	thankfulness
1251391355135225856	2020 (24 Mar - 30 Apr)	home-based work	thankfulness
1251411888967168000	2020 (24 Mar - 30 Apr)	ornamental and vegetable gardening	uplifts
1251461563309084677	2020 (24 Mar - 30 Apr)	socialising and leisure activities	
1251512732861546496	2020 (24 Mar - 30 Apr)	wildlife-friendly activities	
1251572388430479361	2020 (24 Mar - 30 Apr)	wildlife-friendly activities	
1253067787146334208	2020 (24 Mar - 30 Apr)	socialising and leisure activities	contemplation
1253069168972386305	2020 (24 Mar - 30 Apr)	socialising and leisure activities	providing hope
1253074262182760454	2020 (24 Mar - 30 Apr)	wildlife-friendly activities	
1253114637102592001	2020 (24 Mar - 30 Apr)	socialising and leisure activities	
1253118417357484033	2020 (24 Mar - 30 Apr)	ornamental and vegetable gardening	providing hope
1253127130247987207	2020 (24 Mar - 30 Apr)	socialising and leisure activities	
1253184435068993536	2020 (24 Mar - 30 Apr)	home-based work	
1253201033993560065	2020 (24 Mar - 30 Apr)	ornamental and vegetable gardening	
1253202891214643200	2020 (24 Mar - 30 Apr)	ornamental and vegetable gardening	
1253206986117840898	2020 (24 Mar - 30 Apr)	wildlife-friendly activities	
1253209380092338176	2020 (24 Mar - 30 Apr)	wanting a garden	
1253209721915441152	2020 (24 Mar - 30 Apr)	socialising and leisure activities	

1253214209925697537	2020 (24 Mar - 30 Apr)	ornamental and vegetable gardening	uplifts
1253221316364308482	2020 (24 Mar - 30 Apr)	socialising and leisure activities	
1253243210425606145	2020 (24 Mar - 30 Apr)	socialising and leisure activities	thankfulness
1253244022891712512	2020 (24 Mar - 30 Apr)	ornamental and vegetable gardening	
1253244365037867008	2020 (24 Mar - 30 Apr)	wildlife-friendly activities	
1253249329428598784	2020 (24 Mar - 30 Apr)	home-based work	thankfulness
1253249753502101504	2020 (24 Mar - 30 Apr)	ornamental and vegetable gardening	
1253259931383406592	2020 (24 Mar - 30 Apr)	ornamental and vegetable gardening	
1253264229945221121	2020 (24 Mar - 30 Apr)	ornamental and vegetable gardening	mitigation of restlessness
1253275145600409600	2020 (24 Mar - 30 Apr)	home-based work	
1253276512306040833	2020 (24 Mar - 30 Apr)	socialising and leisure activities	
1253277100716576768	2020 (24 Mar - 30 Apr)	socialising and leisure activities	
1253277801945485313	2020 (24 Mar - 30 Apr)	socialising and leisure activities	providing hope
1253285162844028928	2020 (24 Mar - 30 Apr)	wildlife-friendly activities	
1253286890557628417	2020 (24 Mar - 30 Apr)	home-based work	
1253298103354044418	2020 (24 Mar - 30 Apr)	socialising and leisure activities	
1253300384459866112	2020 (24 Mar - 30 Apr)	wanting a garden	
1253303114050011138	2020 (24 Mar - 30 Apr)	socialising and leisure activities	contemplation
1253304152865865729	2020 (24 Mar - 30 Apr)	socialising and leisure activities	thankfulness
1253304450271363073	2020 (24 Mar - 30 Apr)	wildlife-friendly activities	thankfulness
1253307121199955968	2020 (24 Mar - 30 Apr)	wildlife-friendly activities	
1253312081803255813	2020 (24 Mar - 30 Apr)	socialising and leisure activities	
1253314688965922817	2020 (24 Mar - 30 Apr)	socialising and leisure activities	
1253316475932344320	2020 (24 Mar - 30 Apr)	socialising and leisure activities	thankfulness
1253316729033261058	2020 (24 Mar - 30 Apr)	socialising and leisure activities	
1253319248765579265	2020 (24 Mar - 30 Apr)	socialising and leisure activities	
1253321266456489984	2020 (24 Mar - 30 Apr)	wildlife-friendly activities	
1253321732330381313	2020 (24 Mar - 30 Apr)	socialising and leisure activities	

1253932189927997442	2020 (24 Mar - 30 Apr)	wildlife-friendly activities	
1253934490424147968	2020 (24 Mar - 30 Apr)	socialising and leisure activities	
1253998724403073026	2020 (24 Mar - 30 Apr)	ornamental and vegetable gardening	
1254001127886118914	2020 (24 Mar - 30 Apr)	ornamental and vegetable gardening	
1254049743799488513	2020 (24 Mar - 30 Apr)	wildlife-friendly activities	
1254050907995738112	2020 (24 Mar - 30 Apr)	ornamental and vegetable gardening	
1254109295358205952	2020 (24 Mar - 30 Apr)	wildlife-friendly activities	
1254110276741447681	2020 (24 Mar - 30 Apr)	socialising and leisure activities	contemplation
1254311449469399043	2020 (24 Mar - 30 Apr)	socialising and leisure activities	
1254320192353636353	2020 (24 Mar - 30 Apr)	socialising and leisure activities	
1254352682933944324	2020 (24 Mar - 30 Apr)	socialising and leisure activities	
1254354844816740352	2020 (24 Mar - 30 Apr)	wildlife-friendly activities	
1254409955542786050	2020 (24 Mar - 30 Apr)	socialising and leisure activities	uplifts
1254413367701364737	2020 (24 Mar - 30 Apr)	wildlife-friendly activities	
1254472820500901888	2020 (24 Mar - 30 Apr)	ornamental and vegetable gardening	
1254475010946879489	2020 (24 Mar - 30 Apr)	DIY	
1254664645790613504	2020 (24 Mar - 30 Apr)	ornamental and vegetable gardening	
1254668034427535360	2020 (24 Mar - 30 Apr)	DIY	
1254727516385955846	2020 (24 Mar - 30 Apr)	socialising and leisure activities	uplifts
1254739496211558401	2020 (24 Mar - 30 Apr)	socialising and leisure activities	
1255024001400868869	2020 (24 Mar - 30 Apr)	wildlife-friendly activities	
1255033258993205248	2020 (24 Mar - 30 Apr)	socialising and leisure activities	
1255075680502628352	2020 (24 Mar - 30 Apr)	socialising and leisure activities	
1255086043025596417	2020 (24 Mar - 30 Apr)	ornamental and vegetable gardening	
1255137609547788301	2020 (24 Mar - 30 Apr)	socialising and leisure activities	
1255158570867400706	2020 (24 Mar - 30 Apr)	wildlife-friendly activities	
1255210229379276801	2020 (24 Mar - 30 Apr)	socialising and leisure activities	uplifts

1255224645646725121	2020 (24 Mar - 30 Apr)	wildlife-friendly activities	
1255406071495131140	2020 (24 Mar - 30 Apr)	socialising and leisure activities	
1255471330914377728	2020 (24 Mar - 30 Apr)	socialising and leisure activities	thankfulness
1255491361110929410	2020 (24 Mar - 30 Apr)	ornamental and vegetable gardening	
1255516004098113541	2020 (24 Mar - 30 Apr)	ornamental and vegetable gardening	
